# Technostress and its associated factors: Burnout and fatigue among Malaysian healthcare workers (HCWs) in state hospitals

**DOI:** 10.1371/journal.pone.0319506

**Published:** 2025-03-17

**Authors:** Nor Asiah Muhamad, Nur Hasnah Ma’amor, Nurul Hidayah Jamalluddin, Izzah Athirah Rosli, Fatin Norhasny Leman, Tengku Puteri Nadiah Tengku Baharudin Shah, Nurul Syazwani Misnan, Norni Abdullah, Mohammad Zabri Johari, Norliza Chemi, Norashikin Ibrahim, on behalf of the Member of Technostress Study

**Affiliations:** 1 Sector for Evidence Based Healthcare, National Institutes of Health, Ministry of Health Malaysia, Setia Alam, Selangor, Malaysia; 2 Department of Psychiatry & Mental Health, Hospital Tengku Ampuan Rahimah, Ministry of Health Malaysia, Klang, Selangor, Malaysia; 3 Health Behavior Research Institute, National Institutes of Health, Ministry of Health Malaysia, Setia Alam, Selangor, Malaysia; 4 Department of Psychiatry & Mental Health, Hospital Kajang, Ministry of Health Malaysia, Kajang, Selangor, Malaysia,; 5 National Centre of Excellence for Mental Health, Ministry of Health Malaysia, Cyberjaya, Selangor, Malaysia; University of Hafr Al-Batin, SAUDI ARABIA

## Abstract

**Background:**

Technostress is defined as a psychological state associated with the increased usage of advanced computer technologies on a daily basis. It is also defined as an anxiety feeling or mental strain due to excessive exposure or involvement with technologies.

**Aim:**

This study aimed to determine the level of technostress associated with burnout and fatigue among healthcare workers (HCWs) in the state hospitals of Malaysia.

**Methods:**

A cross-sectional study was conducted from September 2022 to November 2023 among HCWs working in the 15-state hospitals in Malaysia. A standardized questionnaire was distributed among the HCWs in the state hospitals in Malaysia. The questionnaire contains information on socio-demography and topic-specific scales on technostress, burnout and fatigue.

**Results:**

A total of 1620 HCWs were included in the analysis, of which 244 (15%) have high level of technostress, 1089 (67%) have moderate technostress, and 287 (18%) have low technostress. Burnout, and fatigue were significantly associated with technostress. HCWs with moderate burnout were less likely to have high technostress compared to those with high burnout (B =  -0.993, 95% CI; 0.231 - 0.594; p <  0.001). Those with moderate fatigue were less likely to have high technostress (B =  -3.844, 95% CI; 0.003 - 0.162; p <  0.001) compared to those with high fatigue.

**Conclusions:**

This study found that majority of the HCWs have moderate level of technostress. Technostress has become more common after the COVID-19 pandemic in 2020 drastically altered working conditions and made remote work using information and communication technologies (ICT) a necessity rather than a luxury. Mitigation measures and programs that include psychological support for individuals who are struggling with the technostress and burnout are needed to overcome this issue.

## Introduction

The usage of information and communication technology (ICT) has increased dramatically in recent years. Working life has become much more computerized since the Movement Control Order (MCO) was put into place to stop the spread of COVID-19 [1]. The use of teleconferencing and online meetings using various technology applications have been expanded largely in education [2], healthcare [3], and business sectors [4]. For example, zoom, a video conferencing app, has seen a huge growth in usage, with roughly 10 million people using the app daily in December 2019, rising to 200 million users in March 2020, and 300 million users in April 2020 [5–6]. The increased use of internet services from 40% to 100% was seen and the usage of video conferencing applications such as Zoom, and Google Meet has seen a 10-fold increased [1] compared to pre-pandemic period. Although the benefits of technology have seen to increase the efficacy and effectiveness of work productivity, it also may lead to technostress among employees [7–8].

Technostress is defined as a psychological state associated with the increased usage of advanced computer technologies on a daily basis [9]. It arises from the negative effects of contemporary technology that lead to addiction and stress [10]. Furthermore, it is also defined as an anxiety feeling or mental strain due to excessive exposure or involvement with technologies [11]. In short, technostress refers to innate fear arising among employees when using new technologies [12]. Another study defined technostress as perceived stress involving ICT use at work [13]. Individuals working in healthcare sectors are among professional employees who are prone to develop technostress [14] due to their intense duties. As healthcare workers (HCWs) who constantly need to deal with difficult situations such as treating patients’ injuries, overcoming the death of patients and continuously delivering care as well as services to patients, they are also getting a burden from the need to use technologies for virtual meetings, presentations and patient follow-up [14] as most of the hospital using technologies to keep their patients’ health information. It was reported that 33% of Egyptian medical staff perceive a high level of technostress [15]. It was also revealed that managers working in healthcare sectors were more likely to suffer from technostress compared to managers in other sectors [13]. A recent study in Switzerland reported healthcare professionals had moderate technostress in their daily work and different healthcare departments experienced different levels of technostress [16].

Perceived stress related to work, depressive disorders or burnout is reported to be associated with mental illness [17] and had been regarded as an occupational hazard. The World Health Organization, WHO (2019), defined burnout as a syndrome resulting from unmanageable perceived stress at the workplace [18]. It can be characterized by feelings of exhaustion or depletion of energy that increased feelings of negativism and reduced work efficiency. Nowadays, burnout is recognized as evidence of a person’s mental well-being [18]. Previously, technology had not been addressed as a source of work stress. However, this has changed with the digitalization transformation in work organization. Gradually, technology has become a contributing factor to work-related stress and may impair mental health well-being including burnout and digital exhaustion [19]. A review reported burnout and exhaustion were associated with high perceived technostress among working adults [20]. According to a study by Spataro (2020), video conferencing is more exhausting than face-to-face meetings since it needs constant attention [21]. Similarly, another study reported technostress was associated with increased degrees of burnout, digital exhaustion, decreased work engagement and efficacy among computer professionals as well as end users [22].

Accurate data is critical to HCWs in order to deliver the best results mostly on patients’ treatment. Therefore, the ubiquitous digital technology has led to implementation of the Health Information Systems (HIS), a system designed to manage patient’s data systematically which is in itself is continuously evolving. Additionally, today’s new norm in conducting virtual meetings, presentations, workshops and training has caused a significant increase in involvement with technology [23]. This has led to concerns on work-related stress perceived to be due to technology among employees. Most studies conducted on technostress uses survey methods and there are limited number of studies using practical experiment method. Despite using different methods, the finding showed similar results. For example, study conducted in Sweden also describe the negative aspects of digital communication and poor used experience of ICT system as technostress creators among HCWs [24]. Similar finding was observed in survey methods perform in Egypt [15].

Technostress and its impacts had been studied in many different sectors and professions, such as among academic staff of higher institutions [11], computer professionals [22], managers [13], school teachers [25], healthcare professionals [16], medical staff and students [15] as well as among academic librarians [26]. Studies on technostress and its impact on mental health are more limited especially among HCWs. The HCWs role has long been regarded as part of stressor based upon the physical labour, human suffering, long work hours, staffing, and interpersonal relationships that are central to their work. Stress, burnout and fatigue remain the significant concerns among HCWs, affecting both individuals and organizations. In order to better comprehend the consequences of technostress and improve risk management of the ubiquitous technology at work, it is seen to be essential to look at the degree of technostress and its effects on workers’ mental health, particularly among healthcare personnel. Therefore, this study aimed to determine the level of technostress and its associated factors among HCWs in the state hospitals of Malaysia.

## Materials and methods

### Study design and ethics

A cross-sectional study was conducted from September 19, 2022 to November 16, 2023 among HCWs at the 15 state hospitals in Malaysia. The hospitals were selected through a stratified random sampling. This study was approved by the Medical Research & Ethic Committee (MREC), Ministry of Health Malaysia (NMRR ID-22-00915-SC3 (IIR)). Written informed consents were obtained prior to conducting this study.

### Study participants

The respondents in this study were HCWs working in 15 state hospitals in Malaysia namely Hospital Tengku Ampuan Rahimah Klang, Selangor, Hospital Kajang, Selangor, Hospital Kuala Lumpur, Hospital Raja Perempuan Zainab II, Kelantan, Hospital Sultanah Nur Zahirah, Terengganu, Hospital Tengku Ampuan Afzan, Pahang, Hospital Sultanah Aminah, Johor, Hospital Melaka, Hospital Tuanku Jaafar, Negeri Sembilan, Hospital Tuanku Fauziah, Perlis, Hospital Sultanah Bahiyah, Kedah, Hospital Raja Permaisuri Bainun, Perak, Hospital Umum Sarawak and Hospital Queen Elizabeth, Sabah. The HCWs were eligible if they worked in the index hospital for a minimum of two years. The included categories of HCWs were doctors, nurses, paramedics, allied health professionals such as radiographer and audiologist, administrative staff and pharmacists. All the HCWs from the 15 included state hospitals were identified by their respective hospital administrators or the employee list from the human resource department. The eligible HCWs were selected through simple random sampling using the registration list and they were invited to participate in this study via email. Only HCWs who agreed to participate were given a link to respond to the questionnaire via email, social platform such as WhatsApp or the organization’s intranet. Other HCWs who declined to participate were asked to reply to the email and informed the investigator that they declined to participate. A self-administered survey was used for this study. Prior to the participants recruitment, they were called to each hospital assembly hall for briefing regarding the study and written informed consent was distributed among all of them.

### Instruments

The constructed questionnaire used in this study included items on sociodemographic profiles, usage on the Internet and electronic devices, assessment on burnout and fatigue, as well as technostress. The questionnaire was in English language therefore, the questionnaire was given to the HCWs who can understand English.

#### Sociodemographic profiles.

Items on the sociodemographic profiles included age, gender, race, education level, occupation, year of service in healthcare sectors, and monthly income.

#### Usage on the internet and electronic devices.

Items on the usage of the Internet and electronic devices included average condition of mobile phone network reception at the current hospital, average condition of hospital’s internet connection, and hours spent on electronic devices for working and leisure purposes.

#### Burnout.

The degree of burnout was assessed using the Oldenburg Burnout Inventory (OLBI) questionnaire, adapted from Demerouti and Nachreiner (1989) [27]. The scale consists of 16 items rated on a four-point scale (1 =  strongly agree, 2 =  agree, 3 =  disagree, 4 =  strongly disagree). This scale had been used to assess level of burnout among different professions, including therapists [28], construction workers [29], Swedish healthcare workers [30], employees working in human services, industrial and transportation sectors [31]. This scale had been validated among medical students in Malaysia by Mahadi et al. (2018) with Cronbach’s alpha of the factors ranging from 0.74 to 0.80, indicating a good reliability [32]. The mean score was calculated to determine the degree of burnout and has been categorized into three levels (low: 1.62 and below, moderate: 1.63 – 2.67 and high: 2.68 and above) [28].

#### Fatigue.

The degree of fatigue will be measured using the Zoom Exhaustion & Fatigue scale (ZEF scale) [33]. The scale consists of five categories of fatigue: (i) general, (ii) social, (iii) emotional, (iv) visual, and (v) motivational. The scale has a total of 15 items rated on five-point Likert scale (1 =  not at all, 2 =  slightly, 3 =  moderately, 4 =  very, 5 =  extremely), except for two frequency questions which are marked with asterisks (1 =  never, 2 =  rarely, 3 =  sometimes, 4 =  often, 5 =  always) [33–34]. The scale was developed by Fauville et al. (2021) with Cronbach’s alpha of more than 0.8 for each of the five constructs of fatigue [33]. The ZEF score ranges from 15 (less fatigue) to 75 (more fatigue).

#### Technostress.

The determination on the level of technostress in this study was adapted from the scale developed by Tarafdar et al. (2007) [8]. The contents use to measure technostress resemble the stressors use in measuring occupational stress [35–36]. The scale is composed of 23 items, rated on a five-point Likert scale ranging from 1 (strongly disagree) to 5 (strongly agree) [3]. The items are grouped into five factors of technostress: (i) Techno-overload: Measures the respondents’ agreement whether the technology used has changed their work pace, work habits, and workload; (ii) Techno-invasion: Measures the respondents’ agreement on how the technology used has encroached into their personal life; (iii) Techno-uncertainty: Measures the respondents’ agreement whether there were constant changes in the technology used in their workplace; (iv) Techno-complexity: Measures the respondents’ perception towards the complexity of the technology used and the adequacy of their existing technological skills and knowledge; and (v) Techno-insecurity: Measures the respondents’ agreement whether the technology used is threatening their job security [37]. This scale had been used among academic librarians in Malaysian public universities [26]. This scale also had been validated among Egyptian medical staff and students with a Cronbach’s alpha of 0.81 [15]. The mean score was calculated to determine the level of technostress and has been categorized into three levels (low: 1.00 – 2.33, moderate: 2.34 – 3.66 and high: 3.67 – 5.00) [38].

### Statistical analysis

The data was analyzed using SPSS version 22.0 (SPSS Inc., Chicago, IL, USA). Participant characteristics, level of technostress, burnout and fatigue were summarized using descriptive analysis by counts and percentages for categorical variables and mean ±  standard deviation (SD) for continuous variables. Pearson’s Chi-square test was conducted to identify factors (sociodemographic factors, internet and electronic devices exposure factors, burnout, and fatigue) associated with the level of technostress. If any factors were observed with statistically significant, further analysis using multinomial logistic regression was performed to assess the potential factors of technostress by reporting the odd ratios (ORs) and their corresponding 95% confidence intervals (95% CI). A p-value of <  0.05 was considered as statistically significant.

## Results

A total of 1877 HCWs were invited to participate in this study. Of these, a total 1620 HCWs agreed to participate giving rise to 86% response rate. Table 1 shows the percentage distribution of sociodemographic profiles, health background, and internet exposure factors among the HCWs. The mean age of the HCWs was 36.8 years old (SD 7.2), and about 70% were below 40 years old. Nearly 76% were females, and majority of the HCWs in the government setting were Malays (69%). In terms of educational level, about half of the HCWs had their certificate or diploma, and the other half held a degree or higher level of education. Almost 95% of the HCWs were allied health professionals and the remaining were management staff. Most of the HCWs (93%) had an experience in the service of 20 years and below. A little more than half (56%) of the HCWs had a monthly income of RM 4850 and above. About 91% of average mobile phone network reception at the current hospital was good, and 84% of the average hospital’s internet connection was good. Almost 74% and 88% of the HCWs spent 6 hours and below on electronic devices for working and leisure purposes, respectively. Concerning health background factors, approximately 84% of the HCWs had moderate burnout, and about 97% had moderate fatigue.

**Table 1:** Basic characteristics of sociodemographic profiles, factors for internet and electronic devices exposures, burnout, and fatigue among the healthcare workers (N = 1620).

	Frequency, N = 1620	Percentage, %
A. Sociodemographic Profiles		
Age group		
Below 40	1126	69
40 and above	494	30
Gender		
Male	390	24
Female	1230	75
Race		
Malay	1124	69
Non-Malay	496	30
Highest education level		
Certificate/ Diploma	805	49
Degree and higher	815	50
Designation		
Medical & Allied Health staff	1537	94.9
Management staff	83	5.1
Year of service		
20 years and below	1514	93
above 20 years	106	6.5
Monthly income		
Below RM 4850	705	43
RM 4850 and above	915	56
B. Internet & Electronic Devices Exposures		
Average mobile phone network reception at the current hospital		
Good	1473	91
Bad	147	9
Average hospital’s internet connection		
Good	1360	84
Bad	260	16
Hours spent on electronic devices for working purposes		
6 hours and below	1192	74
Above 6 hours	428	26
Hours spent on electronic devices for leisure purposes		
6 hours and below	1429	88
Above 6 hours	191	12
C. Burnout		
Moderate	1359	84
High	261	16
D. Fatigue		
Moderate	1567	97
High	53	3

### Technostress among healthcare workers

**Fig 1:**
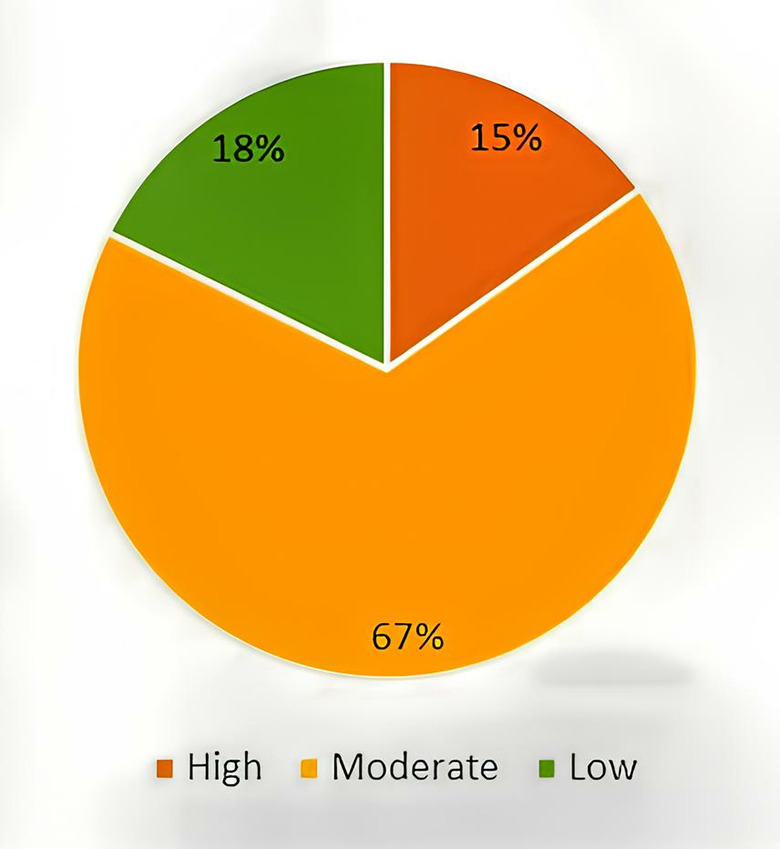
Level of technostress among HCWs.

Of the 1620 HCWs, 244 (15%) have high level of technostress, 1089 (67%) have moderate technostress, and 287 (18%) have low technostress (Fig 1). From the univariate analysis, age (p =  0.001), gender (p =  0.001), education level (p <  0.001), years of service (p =  0.007), and income (p =  0.005) were significantly associated with the level of technostress. In addition, there was also a significant association between hours spent on electronic devices for working purposes and level of technostress (p <  0.001). Burnout, fatigue, and hours spent on devices for working purposes were also found to be significantly associated with technostress (p <  0.001). Table 2 presents the association between sociodemographic, health background, and internet exposure factors with technostress level among HCWs.

**Table 2:** Association between sociodemographic, internet and electronic devices exposure factors, burnout, and fatigue with technostress level among HCWs using univariate analysis.

	Level of Technostress			p-value^a^
	Low N = 287	Moderate N = 1089	High N = 244	
A. Sociodemographic Profiles				
Age group				**0.001**
Below 40	222	753	151	
40 and above	65	336	93	
Gender				**0.001**
Male	93	240	57	
Female	194	849	187	
Race				0.096
Malay	186	759	179	
Non-Malay	101	330	65	
Highest education level				**<0.001**
Certificate/ Diploma	88	575	142	
Degree and higher	199	514	102	
Occupation				0.761
Medical & Allied Health staff	270	1036	231	
Management staff	17	53	13	
Year of service				**0.007**
20 years and below	278	1016	220	
above 20 years	9	73	24	
Income				**0.005**
Below RM 4850	101	487	117	
RM 4850 and above	186	602	127	
B. Internet & Electronic Devices Exposures				
Average mobile phone network reception at the current hospital				0.236
Good	264	994	215	
Bad	23	95	29	
Average hospital’s internet connection				0.706
Good	244	915	201	
Bad	43	174	43	
Hours spent on electronic devices for working purposes				**<0.001**
6 hours and below	234	796	162	
Above 6 hours	53	293	82	
Hours spent on electronic devices for leisure purposes				0.076
6 hours and below	258	966	205	
Above 6 hours	29	123	39	
C. Burnout				
Moderate	252	937	170	**<0.001**
High	35	152	74	
D. Fatigue				
Moderate	286	1061	220	**<0.001**
High	1	28	24	

^a^Pearson’s Chi-square test, significant at p < 0.05 (bold)

Further analysis was performed to determine potential factors of technostress. Table 3 shows results of the multinomial logistic regression modeling of the combined effects of sociodemographic, health background, and internet exposure factors with technostress levels among HCWs. The OR calculations of the model with low level of technostress as the reference category (Table 3), shows that those aged below 40 years old were less likely to have moderate (B =  -0.38, 95% CI; 0.486 - 0.961; p =  0.029) and high technostress (B =  -0.803, 95%; CI 0.288 - 0.696; p <  0.001) compared to those aged 40 years old and above. Factors associated with education level show that those with certificate estimated to be nearly 2.7 times chances to have moderate technostress (B =  0.989; 95% CI:1.886 - 3.836; p <  0.001) and about three times more likely to have high technostress (B =  1.196, 95% CI: 2.082 - 5.249; p <  0.001) as compared to those with degree and above.

**Table 3:** Multinomial logistic regression for factors associated with level of technostress among HCWs.

		Level of Technostress							
		Moderate				High			
		OR	95% CI		p-value^a^	OR	95% CI		p-value^a^
Age group	Below 40	0.684	0.486	0.961	**0.029**	0.448	0.288	0.696	**<0.001**
	40 and above (ref)								
Gender	Male	0.779	0.576	1.053	0.104	0.875	0.575	1.333	0.535
	Female (ref)								
Highest education level	Certificate/ Diploma	2.689	1.886	3.836	**<0.001**	3.306	2.082	5.249	**<0.001**
	Degree and higher (ref)								
Year of service	20 years and below	0.717	0.333	1.542	0.394	0.607	0.253	1.455	0.263
	Above 20 years (ref)								
Income	Below RM 4850	0.914	0.642	1.300	0.616	0.995	0.625	1.583	0.984
	RM 4850 and above (ref)								
Duration use of device for working purposes (hours)	6 hours and below	0.578	0.412	0.812	**0.002**	0.373	0.244	0.572	**<0.001**
	Above 6 hours (ref)								
Burnout	Moderate	0.984	0.655	1.477	0.937	0.373	0.233	0.599	**<0.001**
	High (ref)								
Fatigue	Moderate	0.097	0.013	0.722	**0.023**	0.021	0.003	0.162	**<0.001**
	High (ref)								

The reference category is: Low technostress

^a^significant at p < 0.05 (bold)

In addition, HCWs who spent six hours and below on electronic devices for working purposes were less likely to have moderate (B =  -0.548, 95% CI; 0.412-0.812; p =  0.002) and high technostress (B =  -0.985, 95% CI; 0.244-0.572; p <  0.001) compared to those who spent above six hours. HCWs with moderate burnout were less likely to have high technostress compared to those with high burnout (B =  -0.993, 95% CI; 0.231 -0.594; p <  0.001). Those with moderate fatigue were less likely to have moderate technostress (B =  -2.330, 95% CI; 0.013 -0.722; p =  0.023) and high technostress (B =  -3.844, 95% CI; 0.003 - 0.162; p < 0.001) compared to those with high fatigue.

## Discussion

Technostress is generally referred to as a prominent “dark side” phenomenon that emphasizes the adverse effects of using or applied technology [15,39]. The current study aims to determine the level of technostress and its associated factors among HCWs in the 15 state hospitals in Malaysia. Findings showed that 67% of HCWs had a moderate level of technostress while 15% experienced high technostress level. These findings are similar to a study reported in 2022 which showed that 65% and 33% of the medical staff had moderate and high levels of technostress, respectively [15]. Similar findings were also reported in Iran with the highest level of technostress reported at medium level: 41%, health practitioners or HWs experienced [40]. A different study from Switzerland psychiatric hospitals also reported health professionals had moderate level of technostress [41].

This study also showed age being an important factor contributing to high levels of technostress. One review suggested age differences are related to the ability to adapt to new technologies; although digital technologies have the potential to enhance the quality of working life [42]. The review reported older adults perceived ICT as more difficult to use and face challenges in adapting and utilizing technology in their workplace. Similar findings were reported by Thunder and team whereby most healthcare workers affected with technostress were older workers born in the 1960s and 1970s [43]. A review also reported higher technostress was significantly influenced by age [44].

One study found that HCWs have higher technostress as compared to other professions due to higher skill requirements. However, another study reported that the health professionals or HCWs experienced moderate technostress (mean 39.06, SD 32.54) [16]. Besides treating patients, HCWs are involved in teaching, clinical practice, and research [13,15]. Additionally, another review suggested the level of technostress was also influenced by higher-ranking roles [44]. Furthermore, during COVID-19, telehealth application is also used as a medium of consultation between clinicians and patients due to movement restrictions and this might be the source of technostress problems among HCWs [16].

Globally, electronic health records (EHR) are increasingly being implemented to improve healthcare quality, safety, and efficiency [45]. EHR help to improve patients’ experience of care, and population health, improve clinical decisions, and reduce healthcare costs [45]. However, for some HCWs, it causes burnout and fatigue as they need to key in patients’ information as they are not familiar with the system and causing them to feel incapable to manage their use of ICT [46], contributing to the occurrence of technostress [47]. Integration of the latest technology as most have in the state hospitals in Malaysia are currently changing their way of keeping patients’ health records, i.e., transferring data from hard to soft copy or using electronic health records, service delivery, accuracy in information management and resource optimization [48]. This contributed to increased hours spent on devices for working purposes leading to technostress, burnout, fatigue, increased stress, impacting individual well- being, disrupting work environment, job performance and organizational outcomes [49–50]. Additionally, working in rural areas or smaller towns are other factors that contribute to the higher likelihood of technostress among HCWs mainly to the doctors [51].

In this study, HCWs with a high level of technostress are associated with burnout. Previous study had suggested that HCWs especially clinicians seem to be at particular risk for burnout [52] that might be appears due to under individual constant stress. Furthermore, our finding is similar to another study performed in Switzerland 2021 concluded that technostress is associated with long-term consequences for staff, especially those with burnout symptoms that are linked to depressive mood and anxiety symptoms [16]. Interestingly, the paper stated that further digitization in hospitals is expected to have an increasing impact on the technostress experience [16].

Besides burnout, fatigue is also associated with technostress which might be due to tiredness and exhaustion because of the use of ICT [53] as well as the access to the internet [34]. This fatigue form of computer-mediated communication exhaustion caused by cognitive over-exertion that can lead to stress during and after videoconferencing [34]. It can further adversely affect their work, for example, non-focusing or having concentration problems, biasing their judgments on treating their patients, and decreasing the work performance of the HCWs [54]. This can be observed from this study where both moderate and high technostress level is associated with fatigue.

## Strength and limitations

This study has some limitations that should be addressed. Firstly, participants involved in this study were pre-identified through the employee list, thus the selection bias cannot be ruled out. Data might have been biased by the “healthy worker effect”, which might have led us to underestimate technostress and burnout levels. In addition, it might be possible that HCWs who agreed to participate in this study were healthy enough to work and were overrepresented. Secondly, this study does not rule out previous mental health issues that were experienced by HCWs, which might affect self-reporting and confounding effects of burnout and technostress. Thirdly, out of 15 hospitals included in this study, 3 hospitals are using the EHR while other hospital used manual records. Since most of hospitals are still manual records, therefore we did not perform any analysis focusing on the hospital using EHR versus manual records.

## Conclusion

In this study, findings showed that majority of HCWs have moderate levels of technostress. The association between technostress and sociodemographic, fatigue and burnout were also observed. Since COVID-19 pandemic hit globally in 2020, it caused dramatic changes to working environments and remote work using ICTs became a need rather than a luxury. This led to the technostress and also burnout to the HCWs that has been described by scientists as the dark side of technology use. Mitigation measures and programs that include psychological support for individuals who are struggling with the technostress and burnout are needed to overcome this issue. These programs should include training in creating good networks, use of smart devices, and IT support teams for HCWs as cornerstones to overcoming technology. In short, all the efforts to prevent or mitigate technostress need to be reinforced through involvement, literacy and technical support from the hospital, team and family members.

## Supporting information

SI Appendix Raw data. (PDF)
