# Supplementary material for: Technostress and its associated factors: Burnout and fatigue among Malaysian healthcare workers (HCWs) in state hospitals
Source: PLoS One. 2025 Mar 17;20(3):e0319506. doi: 10.1371/journal.pone.0319506 (PMC11913267; doi:10.1371/journal.pone.0319506)
Supplement: SI Appendix — (PDF) [file pone.0319506.s001.pdf]

1. Number	2. Profession	3. Gender	4. Ethnicity	5. Highest Level of education
1	Doctors	Male	Malay	Degree
2	Paramedic	Female	Malay	Diploma
3	Doctors	Female	Malay	Degree
4	Paramedic	Female	Bumiputera Sabah & Sarawak	Diploma
5	Doctors	Female	Chinese	Degree
6	Doctors	Female	Malay	Degree
7	Doctors	Male	Malay	Degree
8	Doctors	Male	Malay	Degree
9	Doctors	Female	Malay	Degree
10	Doctors	Female	Malay	Degree
11	Paramedic	Female	Malay	Diploma
12	Doctors	Female	Malay	Degree
13	Doctors	Male	Chinese	Degree
14	Administration staff	Female	Malay	Degree
15	Doctors	Female	Chinese	Degree
16	Doctors	Male	Indian	Degree
17	Doctors	Female	Malay	Degree
18	Paramedic	Female	Indian	Diploma
19	Paramedic	Female	Bumiputera	Diploma
20	Paramedic	Female	Malay	Diploma
21	Paramedic	Female	Malay	Diploma
22	Paramedic	Female	Malay	Diploma
23	Paramedic	Female	Malay	Diploma
24	Paramedic	Female	Malay	Diploma
25	Paramedic	Female	Malay	Diploma
26	Paramedic	Female	Malay	Diploma
27	Paramedic	Female	Malay	Diploma
28	Paramedic	Female	Malay	Diploma
29	Paramedic	Female	Malay	Diploma
30	Paramedic	Female	Malay	Diploma
31	Paramedic	Female	Indian	Diploma
32	Paramedic	Female	Malay	Diploma
33	Paramedic	Female	Malay	Diploma
34	Paramedic	Female	Malay	Diploma
35	Paramedic	Female	Indian	Diploma
36	Paramedic	Female	Malay	Diploma
37	Paramedic	Male	Malay	Diploma
38	Paramedic	Female	Malay	Diploma
39	Paramedic	Female	Malay	Diploma

40	Doctors	Female	Malay	Degree
41	Paramedic	Female	Malay	Diploma
42	Doctors	Male	Malay	Degree
43	Paramedic	Female	Malay	Diploma
44	Paramedic	Female	Malay	Diploma
45	Doctors	Male	Malay	Degree
46	Paramedic	Female	Malay	Diploma
47	Paramedic	Female	Malay	Diploma
48	Paramedic	Female	Malay	Diploma
49	Doctors	Male	Indian	Degree
50	Paramedic	Female	Malay	Diploma
51	Paramedic	Female	Malay	Diploma
52	Paramedic	Female	Chinese	Diploma
53	Paramedic	Female	Malay	Diploma
54	Paramedic	Female	Malay	Diploma
55	Paramedic	Female	Malay	Diploma
56	Paramedic	Female	Malay	Diploma
57	Paramedic	Male	Malay	Diploma
58	Paramedic	Female	Malay	Diploma
59	Paramedic	Female	Malay	Degree
60	Paramedic	Female	Malay	Diploma
61	Paramedic	Female	Malay	Diploma
62	Paramedic	Female	Malay	Diploma
63	Paramedic	Female	Malay	Diploma
64	Technical staff	Female	Malay	Diploma
65	Paramedic	Female	Malay	Diploma
66	Paramedic	Female	Malay	Diploma
67	Paramedic	Female	Malay	Diploma
68	Paramedic	Female	Malay	Diploma
69	Paramedic	Female	Malay	Diploma
70	Paramedic	Female	Malay	Diploma
71	Paramedic	Female	Malay	Diploma
72	Paramedic	Female	Malay	Diploma
73	Paramedic	Female	Malay	Diploma
74	Paramedic	Female	Malay	Diploma
75	Paramedic	Female	Malay	Diploma
76	Paramedic	Female	Malay	Diploma
77	Paramedic	Female	Malay	Diploma
78	Administratio	Female	Malay	Diploma
79	Paramedic	Female	Malay	Diploma
80	Paramedic	Female	Malay	Diploma
81	Paramedic	Male	Malay	Diploma
82	Administratio	Male	Malay	Diploma
83	Paramedic	Female	Malay	Diploma

84	Paramedic	Female	Malay	Diploma
85	Paramedic	Female	Malay	Diploma
86	Paramedic	Female	Malay	Diploma
87	Paramedic	Female	Malay	Diploma
88	Paramedic	Female	Malay	Diploma
89	Doctors	Female	Chinese	Degree
90	Paramedic	Female	Malay	Diploma
91	Paramedic	Female	Indian	Diploma
92	Paramedic	Female	Malay	Degree
93	Paramedic	Female	Malay	Diploma
94	Paramedic	Female	Malay	Diploma
95	Paramedic	Female	Malay	Diploma
96	Paramedic	Female	Malay	Diploma
97	Paramedic	Male	Malay	Diploma
98	Paramedic	Female	Malay	Diploma
99	Paramedic	Female	Malay	Diploma
100	Paramedic	Female	Malay	Diploma
101	Paramedic	Female	Malay	Diploma
102	Paramedic	Female	Malay	Diploma
103	Paramedic	Female	Malay	Diploma
104	Paramedic	Female	Malay	Diploma
105	Paramedic	Female	Malay	Diploma
106	Paramedic	Female	Chinese	Degree
107	Paramedic	Female	Malay	Diploma
108	Paramedic	Female	Malay	Diploma
109	Paramedic	Female	Malay	Diploma
110	Administratio	Female	Malay	Degree
111	Paramedic	Female	Indian	Master
112	Paramedic	Female	Malay	Diploma
113	Paramedic	Male	Malay	Diploma
114	Paramedic	Female	Malay	Diploma
115	Paramedic	Female	Malay	Diploma
116	Paramedic	Female	Indian	Diploma
117	Paramedic	Female	Indian	Diploma
118	Paramedic	Female	Malay	Diploma
119	Paramedic	Female	Malay	Diploma
120	Paramedic	Male	Malay	Diploma
121	Paramedic	Female	Malay	Diploma
122	Paramedic	Female	Malay	Diploma
123	Paramedic	Female	Malay	Diploma
124	Doctors	Female	Malay	Degree
125	Paramedic	Female	Malay	Diploma
126	Paramedic	Female	Malay	Diploma
127	Paramedic	Male	Malay	Diploma

128	Paramedic	Male	Malay	Diploma
129	Paramedic	Female	Malay	Diploma
130	Paramedic	Female	Malay	Diploma
131	Paramedic	Female	Malay	Diploma
132	Paramedic	Female	Malay	Diploma
133	Paramedic	Female	Malay	Diploma
134	Paramedic	Female	Malay	Degree
135	Paramedic	Female	Malay	Degree
136	Paramedic	Female	Malay	Diploma
137	Paramedic	Female	Malay	Diploma
138	Paramedic	Female	Malay	Degree
139	Paramedic	Female	Malay	Diploma
140	Paramedic	Female	Malay	Diploma
141	Paramedic	Female	Malay	Diploma
142	Paramedic	Female	Malay	Diploma
143	Paramedic	Female	Malay	Diploma
144	Paramedic	Female	Malay	Diploma
145	Paramedic	Female	Bumiputera	Diploma
146	Paramedic	Female	Malay	Diploma
147	Paramedic	Female	Malay	Diploma
148	Paramedic	Female	Malay	Diploma
149	Paramedic	Female	Malay	Diploma
150	Paramedic	Male	Malay	Diploma
151	Paramedic	Female	Malay	Diploma
152	Paramedic	Female	Malay	Diploma
153	Paramedic	Female	Malay	Diploma
154	Paramedic	Female	Chinese	Diploma
155	Paramedic	Female	Malay	Diploma
156	Paramedic	Female	Malay	Diploma
157	Paramedic	Female	Malay	Diploma
158	Paramedic	Female	Malay	Diploma
159	Paramedic	Female	Malay	Diploma
160	Paramedic	Female	Malay	Diploma
161	Paramedic	Female	Malay	Diploma
162	Paramedic	Female	Malay	Diploma
163	Paramedic	Female	Malay	Diploma
164	Paramedic	Female	Malay	Diploma
165	Paramedic	Female	Malay	Diploma
166	Paramedic	Male	Malay	Diploma
167	Paramedic	Female	Malay	Diploma
168	Paramedic	Female	Malay	Degree
169	Paramedic	Female	Malay	Diploma
170	Paramedic	Female	Malay	Diploma
171	Paramedic	Female	Malay	Diploma

172	Paramedic	Female	Malay	Diploma
173	Paramedic	Female	Malay	Diploma
174	Paramedic	Female	Malay	Diploma
175	Paramedic	Female	Malay	Diploma
176	Paramedic	Female	Malay	Diploma
177	Paramedic	Female	Malay	Diploma
178	Administratio	Female	Malay	Diploma
179	Paramedic	Female	Malay	Diploma
180	Paramedic	Female	Malay	Diploma
181	Paramedic	Female	Malay	Diploma
182	Paramedic	Female	Malay	Diploma
183	Paramedic	Female	Indian	Degree
184	Paramedic	Female	Indian	Diploma
185	Paramedic	Female	Malay	Diploma
186	Paramedic	Female	Malay	Diploma
187	Paramedic	Female	Malay	Diploma
188	Paramedic	Male	Malay	Diploma
189	Paramedic	Female	Malay	Diploma
190	Paramedic	Male	Malay	Diploma
191	Paramedic	Female	Malay	Diploma
192	Paramedic	Female	Malay	Diploma
193	Paramedic	Female	Malay	Diploma
194	Paramedic	Female	Malay	Degree
195	Paramedic	Female	Malay	Diploma
196	Paramedic	Female	Malay	Diploma
197	Paramedic	Female	Malay	Diploma
198	Paramedic	Female	Malay	Diploma
199	Doctors	Male	Chinese	Master
200	Doctors	Male	Bumiputera	Master
201	Paramedic	Female	Malay	Degree
202	Doctors	Female	Malay	Degree
203	Paramedic	Female	Malay	Diploma
204	Paramedic	Female	Malay	Diploma
205	Doctors	Female	Indian	Degree
206	Paramedic	Female	Malay	Degree
207	Technical staff	Female	Malay	Degree
208	Doctors	Male	Chinese	Master
209	Doctors	Female	Chinese	Master
210	Doctors	Female	Malay	Degree
211	Doctors	Female	Malay	Degree
212	Doctors	Female	Malay	Degree
213	Technical staff	Female	Malay	Degree
214	Doctors	Female	Malay	Master
215	Doctors	Female	Malay	Master

216	Doctors	Female	Malay	Degree
217	Doctors	Male	Indian	Degree
218	Doctors	Male	Indian	Degree
219	Doctors	Female	Indian	Master
220	Doctors	Female	Malay	Master
221	Doctors	Female	Others	PhD / Subspecialty
222	Doctors	Female	Malay	Master
223	Doctors	Male	Malay	PhD / Subspecialty
224	Doctors	Female	Malay	Degree
225	Technical staff	Female	Malay	Master
226	Technical staff	Female	Indian	Degree
227	Technical staff	Female	Malay	Degree
228	Doctors	Female	Malay	Degree
229	Technical staff	Male	Malay	Degree
230	Technical staff	Female	Indian	Degree
231	Paramedic	Female	Chinese	Degree
232	Paramedic	Male	Malay	Diploma
233	Technical staff	Female	Indian	Degree
234	Paramedic	Male	Malay	Diploma
235	Doctors	Female	Malay	Degree
236	Technical staff	Female	Chinese	Degree
237	Technical staff	Female	Chinese	Degree
238	Doctors	Female	Malay	Master
239	Paramedic	Female	Bumiputera	Diploma
240	Paramedic	Male	Malay	Diploma
241	Paramedic	Female	Indian	Degree
242	Administratio	Female	Malay	Diploma
243	Paramedic	Female	Malay	Diploma
244	Paramedic	Female	Malay	Diploma
245	Paramedic	Female	Malay	Diploma
246	Paramedic	Female	Others	Diploma
247	Paramedic	Female	Malay	Diploma
248	Paramedic	Female	Malay	Diploma
249	Paramedic	Female	Indian	Diploma
250	Paramedic	Female	Indian	Diploma
251	Paramedic	Female	Malay	Diploma
252	Paramedic	Female	Malay	Diploma
253	Paramedic	Female	Bumiputera	Diploma
254	Paramedic	Male	Chinese	Diploma
255	Technical staff	Female	Malay	Diploma
256	Paramedic	Female	Malay	Diploma
257	Paramedic	Male	Malay	Diploma
258	Paramedic	Female	Malay	Diploma
259	Paramedic	Female	Malay	Diploma

260	Doctors	Female	Malay	Degree
261	Paramedic	Female	Malay	Diploma
262	Paramedic	Female	Malay	Diploma
263	Administratio	Female	Malay	Diploma
264	Doctors	Male	Malay	PhD / Subspecialty
265	Paramedic	Female	Malay	Diploma
266	Paramedic	Female	Malay	Diploma
267	Paramedic	Female	Malay	Diploma
268	Paramedic	Male	Indian	Degree
269	Paramedic	Female	Malay	Diploma
270	Paramedic	Male	Malay	Diploma
271	Doctors	Female	Chinese	PhD / Subspecialty
272	Technical staff	Female	Bumiputera	Diploma
273	Doctors	Male	Chinese	Master
274	Doctors	Female	Malay	Degree
275	Doctors	Male	Chinese	Degree
276	Paramedic	Male	Indian	Diploma
277	Paramedic	Female	Bumiputera	Diploma
278	Paramedic	Female	Malay	Diploma
279	Doctors	Female	Malay	Degree
280	Paramedic	Male	Others	Diploma
281	Paramedic	Male	Malay	Diploma
282	Technical staff	Female	Chinese	Degree
283	Doctors	Female	Chinese	Degree
284	Doctors	Female	Malay	Degree
285	Technical staff	Female	Chinese	Diploma
286	Paramedic	Female	Malay	Diploma
287	Doctors	Female	Chinese	PhD / Subspecialty
288	Doctors	Male	Malay	Degree
289	Doctors	Male	Malay	Degree
290	Doctors	Female	Indian	Degree
291	Paramedic	Male	Malay	Diploma
292	Paramedic	Female	Malay	Secondary
293	Doctors	Male	Chinese	Degree
294	Paramedic	Female	Malay	Degree
295	Doctors	Male	Malay	Master
296	Doctors	Male	Chinese	Degree
297	Doctors	Female	Malay	Degree
298	Doctors	Male	Chinese	Degree
299	Paramedic	Female	Malay	Degree
300	Paramedic	Female	Malay	Diploma
301	Paramedic	Female	Malay	Diploma
302	Paramedic	Female	Malay	Diploma

303	Paramedic	Female	Malay	Diploma
304	Paramedic	Female	Malay	Diploma
305	Paramedic	Female	Malay	Diploma
306	Paramedic	Female	Malay	Diploma
307	Paramedic	Female	Indian	Degree
308	Paramedic	Female	Malay	Diploma
309	Paramedic	Female	Malay	Master
310	Paramedic	Female	Malay	Diploma
311	Paramedic	Male	Malay	Diploma
312	Paramedic	Female	Malay	Diploma
313	Paramedic	Female	Malay	Diploma
314	Doctors	Female	Malay	Degree
315	Paramedic	Female	Malay	Diploma
316	Doctors	Female	Malay	Master
317	Technical staff	Female	Malay	Diploma
318	Paramedic	Female	Malay	Diploma
319	Paramedic	Female	Malay	Diploma
320	Doctors	Female	Indian	Master
321	Doctors	Female	Malay	PhD / Subspecialty
322	Doctors	Female	Malay	Degree
323	Doctors	Male	Indian	Degree
324	Doctors	Male	Malay	Secondary
325	Doctors	Female	Indian	Degree
326	Paramedic	Male	Malay	Diploma
327	Doctors	Male	Chinese	Degree
328	Doctors	Female	Chinese	Degree
329	Paramedic	Male	Malay	Degree
330	Paramedic	Male	Malay	Diploma
331	Doctors	Female	Bumiputera	Degree
332	Paramedic	Male	Malay	Diploma
333	Doctors	Female	Indian	Degree
334	Paramedic	Female	Malay	Degree
335	Doctors	Female	Chinese	Degree
336	Paramedic	Female	Malay	Diploma
337	Doctors	Male	Malay	Degree
338	Doctors	Female	Malay	Degree
339	Technical staff	Female	Malay	Degree
340	Paramedic	Female	Malay	Diploma
341	Administratio	Male	Malay	Degree
342	Administratio	Female	Malay	Degree
343	Doctors	Female	Chinese	Degree
344	Paramedic	Female	Malay	Diploma
345	Technical staff	Female	Malay	Master
346	Doctors	Male	Malay	Degree

347	Doctors	Male	Indian	PhD / Subspecialty
348	Paramedic	Female	Indian	Diploma
349	Paramedic	Female	Malay	Diploma
350	Paramedic	Female	Malay	Secondary
351	Doctors	Male	Chinese	Degree
352	Doctors	Male	Malay	Degree
353	Paramedic	Female	Malay	Diploma
354	Doctors	Female	Malay	Master
355	Doctors	Male	Malay	Master
356	Paramedic	Female	Malay	Diploma
357	Paramedic	Female	Chinese	Master
358	Paramedic	Male	Malay	Diploma
359	Paramedic	Male	Malay	Diploma
360	Paramedic	Male	Malay	Secondary
361	Paramedic	Female	Malay	Degree
362	Doctors	Male	Malay	Degree
363	Technical staff	Female	Malay	Degree
364	Paramedic	Female	Malay	Diploma
365	Paramedic	Female	Malay	Diploma
366	Doctors	Male	Indian	Master
367	Paramedic	Female	Malay	Diploma
368	Administratio	Female	Malay	Secondary
369	Paramedic	Female	Malay	Diploma
370	Paramedic	Female	Malay	Diploma
371	Paramedic	Female	Malay	Diploma
372	Doctors	Female	Chinese	Degree
373	Doctors	Female	Malay	Degree
374	Paramedic	Female	Malay	Diploma
375	Doctors	Male	Others	Master
376	Doctors	Male	Chinese	Master
377	Paramedic	Male	Indian	Diploma
378	Paramedic	Female	Malay	Diploma
379	Doctors	Female	Malay	Master
380	Doctors	Male	Indian	Master
381	Paramedic	Female	Malay	Diploma
382	Paramedic	Female	Malay	Diploma
383	Technical staff	Female	Malay	Master
384	Paramedic	Female	Malay	Diploma
385	Technical staff	Female	Malay	Diploma
386	Doctors	Female	Malay	Degree
387	Doctors	Female	Indian	Degree
388	Doctors	Female	Indian	Master
389	Technical staff	Male	Malay	Degree
390	Technical staff	Female	Indian	Degree

391	Technical staff	Female	Malay	Master
392	Doctors	Female	Malay	PhD / Subspecialty
393	Doctors	Female	Chinese	Degree
394	Doctors	Female	Malay	Degree
395	Paramedic	Male	Malay	Diploma
396	Doctors	Female	Malay	Degree
397	Paramedic	Female	Malay	Degree
398	Paramedic	Female	Indian	Diploma
399	Technical staff	Female	Malay	Degree
400	Doctors	Female	Malay	Degree
401	Paramedic	Female	Indian	Master
402	Technical staff	Female	Malay	Degree
403	Paramedic	Male	Chinese	Degree
404	Paramedic	Female	Malay	Master
405	Doctors	Female	Malay	PhD / Subspecialty
406	Doctors	Male	Chinese	Master
407	Technical staff	Female	Chinese	Degree
408	Administratio	Female	Malay	Diploma
409	Paramedic	Male	Bumiputera	Diploma
410	Doctors	Male	Malay	Degree
411	Doctors	Female	Chinese	PhD / Subspecialty
412	Administratio	Female	Bumiputera	Secondary
413	Doctors	Female	Indian	Degree
414	Doctors	Female	Malay	Degree
415	Doctors	Male	Malay	Degree
416	Paramedic	Female	Malay	Diploma
417	Doctors	Female	Indian	Degree
418	Doctors	Female	Malay	Degree
419	Doctors	Female	Malay	Degree
420	Doctors	Female	Chinese	Master
421	Doctors	Female	Malay	Degree
422	Paramedic	Female	Bumiputera	Diploma
423	Doctors	Male	Malay	Degree
424	Technical staff	Female	Bumiputera	Master
425	Doctors	Female	Malay	Degree
426	Paramedic	Female	Malay	Diploma
427	Paramedic	Female	Bumiputera	Diploma
428	Paramedic	Female	Bumiputera	Degree
429	Doctors	Female	Malay	Degree
430	Paramedic	Female	Bumiputera	Diploma
431	Paramedic	Female	Malay	Degree
432	Paramedic	Male	Malay	Degree
433	Paramedic	Female	Malay	Diploma
434	Paramedic	Male	Malay	Diploma

435	Doctors	Male	Others	Degree
436	Doctors	Female	Malay	Master
437	Paramedic	Male	Bumiputera	Degree
438	Doctors	Female	Indian	PhD / Subspecialty
439	Doctors	Male	Chinese	Degree
440	Doctors	Female	Bumiputera	Degree
441	Doctors	Female	Malay	Degree
442	Doctors	Male	Chinese	Master
443	Doctors	Female	Malay	Degree
444	Paramedic	Female	Malay	Degree
445	Doctors	Female	Chinese	Master
446	Doctors	Male	Malay	Degree
447	Doctors	Female	Malay	PhD / Subspecialty
448	Technical staff	Female	Chinese	Degree
449	Doctors	Female	Chinese	PhD / Subspecialty
450	Technical staff	Female	Malay	Degree
451	Doctors	Female	Malay	Degree
452	Paramedic	Male	Bumiputera	Diploma
453	Paramedic	Female	Malay	Diploma
454	Paramedic	Female	Malay	Degree
455	Administratio	Male	Malay	Secondary
456	Administratio	Female	Malay	Diploma
457	Administratio	Female	Malay	Secondary
458	Paramedic	Male	Malay	Degree
459	Administratio	Female	Malay	Diploma
460	Paramedic	Male	Malay	Master
461	Administratio	Female	Malay	Secondary
462	Paramedic	Female	Indian	Degree
463	Paramedic	Female	Malay	Diploma
464	Paramedic	Female	Chinese	Diploma
465	Technical staff	Female	Malay	Degree
466	Paramedic	Female	Bumiputera	Diploma
467	Doctors	Female	Malay	Degree
468	Doctors	Female	Chinese	Degree
469	Paramedic	Female	Malay	Diploma
470	Administratio	Female	Malay	Diploma
471	Paramedic	Female	Malay	Diploma
472	Paramedic	Female	Malay	Diploma
473	Paramedic	Female	Malay	Diploma
474	Paramedic	Female	Malay	Diploma
475	Paramedic	Female	Malay	Diploma
476	Paramedic	Female	Malay	Diploma
477	Administratio	Female	Malay	Secondary
478	Paramedic	Female	Malay	Diploma

479	Doctors	Male	Chinese	Master
480	Doctors	Male	Chinese	Master
481	Paramedic	Female	Malay	Diploma
482	Doctors	Female	Malay	PhD / Subspecialty
483	Paramedic	Female	Malay	Degree
484	Doctors	Female	Indian	Master
485	Technical staff	Female	Malay	Degree
486	Paramedic	Female	Malay	Diploma
487	Doctors	Male	Malay	Degree
488	Technical staff	Male	Chinese	Master
489	Doctors	Female	Malay	Degree
490	Doctors	Female	Indian	Degree
491	Doctors	Female	Chinese	Degree
492	Doctors	Male	Indian	Degree
493	Paramedic	Female	Malay	Diploma
494	Paramedic	Female	Malay	Degree
495	Paramedic	Female	Malay	Diploma
496	Paramedic	Female	Chinese	Diploma
497	Paramedic	Female	Bumiputera	Diploma
498	Paramedic	Female	Malay	Secondary
499	Paramedic	Female	Others	Diploma
500	Paramedic	Female	Bumiputera	Secondary
501	Paramedic	Female	Bumiputera	Diploma
502	Paramedic	Female	Bumiputera	Degree
503	Paramedic	Male	Malay	Diploma
504	Paramedic	Female	Indian	Diploma
505	Paramedic	Female	Malay	Diploma
506	Paramedic	Female	Malay	Diploma
507	Paramedic	Male	Malay	Diploma
508	Doctors	Female	Malay	Degree
509	Paramedic	Female	Bumiputera	Diploma
510	Paramedic	Female	Malay	Diploma
511	Technical staff	Male	Bumiputera	Diploma
512	Paramedic	Male	Malay	Diploma
513	Paramedic	Female	Others	Diploma
514	Paramedic	Male	Bumiputera	Diploma
515	Paramedic	Female	Bumiputera	Degree
516	Paramedic	Female	Bumiputera	Diploma
517	Paramedic	Female	Bumiputera	Diploma
518	Paramedic	Female	Malay	Diploma
519	Technical staff	Female	Indian	Degree
520	Doctors	Female	Indian	PhD / Subspecialty
521	Doctors	Male	Chinese	Degree
522	Doctors	Female	Chinese	Degree

523	Technical staff	Female	Malay	Diploma
524	Technical staff	Male	Malay	Diploma
525	Technical staff	Female	Malay	Degree
526	Technical staff	Female	Malay	Diploma
527	Paramedic	Female	Malay	Diploma
528	Paramedic	Female	Bumiputera	Diploma
529	Paramedic	Female	Bumiputera	Diploma
530	Paramedic	Female	Bumiputera	Degree
531	Technical staff	Female	Chinese	Degree
532	Technical staff	Female	Malay	Degree
533	Paramedic	Female	Bumiputera	Diploma
534	Paramedic	Female	Bumiputera	Diploma
535	Paramedic	Female	Bumiputera	Diploma
536	Doctors	Male	Chinese	Master
537	Paramedic	Female	Bumiputera	Diploma
538	Paramedic	Female	Malay	Diploma
539	Paramedic	Female	Bumiputera	Diploma
540	Paramedic	Female	Bumiputera	Diploma
541	Technical staff	Female	Malay	Degree
542	Paramedic	Female	Bumiputera	Diploma
543	Paramedic	Female	Bumiputera	Diploma
544	Paramedic	Female	Bumiputera	Diploma
545	Paramedic	Female	Malay	Diploma
546	Paramedic	Female	Bumiputera	Diploma
547	Paramedic	Female	Bumiputera	Diploma
548	Paramedic	Female	Bumiputera	Diploma
549	Paramedic	Female	Malay	Diploma
550	Paramedic	Female	Bumiputera	Diploma
551	Paramedic	Female	Bumiputera	Diploma
552	Paramedic	Female	Malay	Diploma
553	Paramedic	Female	Bumiputera	Diploma
554	Paramedic	Female	Bumiputera	Diploma
555	Paramedic	Female	Bumiputera	Diploma
556	Paramedic	Female	Malay	Diploma
557	Paramedic	Female	Bumiputera	Diploma
558	Paramedic	Female	Bumiputera	Diploma
559	Paramedic	Female	Malay	Diploma
560	Paramedic	Female	Malay	Diploma
561	Paramedic	Female	Malay	Diploma
562	Paramedic	Female	Malay	Degree
563	Paramedic	Female	Malay	Diploma
564	Paramedic	Female	Bumiputera	Secondary
565	Paramedic	Female	Bumiputera	Diploma
566	Paramedic	Female	Bumiputera	Diploma

567	Paramedic	Female	Chinese	Diploma
568	Doctors	Female	Malay	Master
569	Paramedic	Female	Bumiputera	Diploma
570	Paramedic	Female	Malay	Diploma
571	Paramedic	Female	Bumiputera	Diploma
572	Paramedic	Female	Malay	Diploma
573	Paramedic	Female	Bumiputera	Diploma
574	Paramedic	Female	Chinese	Diploma
575	Paramedic	Female	Bumiputera	Diploma
576	Paramedic	Female	Bumiputera	Diploma
577	Paramedic	Female	Bumiputera	Diploma
578	Paramedic	Female	Bumiputera	Diploma
579	Paramedic	Female	Malay	Diploma
580	Paramedic	Male	Bumiputera	Diploma
581	Doctors	Female	Chinese	Degree
582	Doctors	Female	Malay	PhD / Subspecialty
583	Paramedic	Female	Bumiputera	Diploma
584	Paramedic	Female	Malay	Diploma
585	Paramedic	Female	Malay	Degree
586	Doctors	Female	Indian	Master
587	Paramedic	Female	Bumiputera	Diploma
588	Doctors	Female	Chinese	Degree
589	Technical staff	Female	Malay	Degree
590	Doctors	Male	Malay	Degree
591	Technical staff	Female	Malay	Master
592	Administratio	Female	Malay	Diploma
593	Paramedic	Female	Bumiputera	Secondary
594	Doctors	Female	Malay	Master
595	Technical staff	Female	Malay	Degree
596	Doctors	Female	Malay	PhD / Subspecialty
597	Doctors	Male	Malay	Degree
598	Doctors	Female	Malay	Degree
599	Doctors	Male	Indian	Degree
600	Doctors	Female	Indian	Degree
601	Paramedic	Female	Bumiputera	Diploma
602	Doctors	Male	Chinese	Degree
603	Doctors	Female	Malay	Master
604	Doctors	Female	Malay	Degree
605	Paramedic	Female	Bumiputera	Diploma
606	Paramedic	Female	Malay	Secondary
607	Doctors	Female	Malay	Master
608	Doctors	Male	Indian	Degree
609	Paramedic	Female	Bumiputera	Diploma
610	Technical staff	Female	Malay	Master

611	Paramedic	Female	Malay	Diploma
612	Technical staff	Female	Malay	Degree
613	Doctors	Male	Malay	Degree
614	Paramedic	Female	Bumiputera	Diploma
615	Doctors	Male	Malay	Master
616	Paramedic	Female	Bumiputera	Degree
617	Doctors	Female	Malay	Master
618	Doctors	Female	Chinese	Degree
619	Doctors	Male	Malay	PhD / Subspecialty
620	Paramedic	Female	Malay	Master
621	Paramedic	Female	Malay	Diploma
622	Doctors	Male	Malay	Master
623	Paramedic	Female	Bumiputera	Degree
624	Doctors	Female	Malay	Degree
625	Paramedic	Female	Malay	Diploma
626	Paramedic	Female	Malay	Degree
627	Paramedic	Female	Bumiputera	Degree
628	Technical staff	Female	Chinese	Degree
629	Technical staff	Male	Malay	Degree
630	Paramedic	Female	Malay	Master
631	Technical staff	Female	Malay	Master
632	Technical staff	Female	Malay	Degree
633	Paramedic	Female	Malay	Degree
634	Doctors	Female	Malay	Degree
635	Technical staff	Female	Chinese	Degree
636	Doctors	Male	Chinese	Master
637	Paramedic	Male	Malay	Diploma
638	Paramedic	Female	India	Diploma
639	Doctors	Male	Malay	Master
640	Doctors	Female	Malay	Degree
641	Doctors	Male	Malay	Master
642	Paramedic	Female	Malay	Diploma
643	Technical staff	Female	Malay	Master
644	Doctors	Female	Malay	Master
645	Doctors	Female	Malay	Master
646	Doctors	Female	Malay	Degree
647	Doctors	Female	Malay	Degree
648	Technical staff	Male	Malay	Degree
649	Paramedic	Female	Malay	Degree
650	Administratio	Female	Malay	Degree
651	Technical staff	Female	Malay	Degree
652	Administratio	Male	Malay	Diploma
653	Doctors	Female	Malay	Master
654	Doctors	Male	Malay	PhD / Subspecialty

655	Doctors	Female	Malay	Master
656	Paramedic	Female	Malay	Degree
657	Doctors	Female	Malay	Master
658	Doctors	Male	Malay	Master
659	Doctors	Male	Malay	Master
660	Paramedic	Male	Malay	Diploma
661	Doctors	Male	Malay	Degree
662	Doctors	Female	Malay	Degree
663	Doctors	Male	Malay	Master
664	Doctors	Female	India	Degree
665	Doctors	Female	Malay	Degree
666	Doctors	Female	Malay	PhD / Subspecialty
667	Doctors	Female	Chinese	Degree
668	Doctors	Female	Malay	Master
669	Doctors	Female	Malay	Degree
670	Doctors	Male	Chinese	Master
671	Paramedic	Female	Malay	Diploma
672	Doctors	Male	Chinese	Degree
673	Paramedic	Female	Malay	Master
674	Doctors	Male	India	PhD / Subspecialty
675	Doctors	Male	Chinese	Degree
676	Doctors	Female	India	Master
677	Technical staff	Male	Malay	Master
678	Administratio	Female	Malay	Degree
679	Doctors	Female	Malay	Master
680	Doctors	Female	Malay	Degree
681	Paramedic	Female	Malay	Diploma
682	Technical staff	Female	Malay	Master
683	Paramedic	Female	Malay	Diploma
684	Paramedic	Female	Malay	Master
685	Doctors	Female	Malay	Degree
686	Paramedic	Female	Malay	Diploma
687	Doctors	Female	Malay	Master
688	Doctors	Female	India	Degree
689	Paramedic	Female	India	Master
690	Paramedic	Female	Malay	Diploma
691	Paramedic	Female	Malay	PhD / Subspecialty
692	Doctors	Male	Chinese	Master
693	Doctors	Female	Malay	Master
694	Paramedic	Female	Chinese	Degree
695	Paramedic	Female	Malay	Diploma
696	Paramedic	Female	India	Degree
697	Paramedic	Female	Malay	Diploma
698	Paramedic	Male	Malay	Diploma

699	Paramedic	Male	Malay	Diploma
700	Paramedic	Female	Malay	Degree
701	Paramedic	Female	Malay	Diploma
702	Doctors	Female	India	Degree
703	Paramedic	Male	Malay	Diploma
704	Paramedic	Female	Malay	Diploma
705	Doctors	Female	Malay	Degree
706	Doctors	Female	Chinese	Degree
707	Doctors	Female	Malay	Degree
708	Doctors	Female	Chinese	Degree
709	Doctors	Female	Chinese	Master
710	Paramedic	Female	Malay	Diploma
711	Doctors	Female	Chinese	Degree
712	Technical staff	Female	Malay	Diploma
713	Technical staff	Female	Malay	Degree
714	Paramedic	Male	Malay	Diploma
715	Paramedic	Female	India	Diploma
716	Paramedic	Female	India	Degree
717	Administratio	Female	Malay	Diploma
718	Paramedic	Female	Malay	Diploma
719	Doctors	Female	India	Degree
720	Doctors	Male	Chinese	Master
721	Doctors	Female	India	PhD / Subspecialty
722	Paramedic	Female	Bumiputera	Diploma
723	Paramedic	Female	Bumiputera	Diploma
724	Paramedic	Female	Bumiputera	Diploma
725	Doctors	Female	Chinese	Master
726	Doctors	Male	Malay	Master
727	Paramedic	Female	Bumiputera	Diploma
728	Doctors	Female	Bumiputera	Master
729	Paramedic	Female	Bumiputera	Diploma
730	Paramedic	Female	Bumiputera	Diploma
731	Paramedic	Female	Bumiputera	Diploma
732	Doctors	Male	Malay	Master
733	Doctors	Male	Chinese	PhD / Subspecialty
734	Doctors	Male	Chinese	Master
735	Doctors	Male	Chinese	Master
736	Doctors	Female	Malay	Master
737	Technical staff	Female	India	Master
738	Technical staff	Female	Chinese	Degree
739	Doctors	Male	Malay	Degree
740	Paramedic	Female	Malay	Diploma
741	Doctors	Male	Chinese	Diploma
742	Doctors	Female	India	Master

743	Paramedic	Male	Bumiputera	Diploma
744	Doctors	Female	Malay	Degree
745	Paramedic	Female	Malay	Diploma
746	Paramedic	Male	Malay	Diploma
747	Paramedic	Female	Malay	Master
748	Paramedic	Male	Malay	Diploma
749	Doctors	Female	India	Degree
750	Technical staff	Male	Malay	Diploma
751	Technical staff	Female	India	Degree
752	Doctors	Female	Malay	Degree
753	Doctors	Female	Malay	Master
754	Doctors	Female	Malay	Degree
755	Doctors	Male	Malay	Master
756	Doctors	Male	Malay	Degree
757	Paramedic	Male	Malay	Diploma
758	Doctors	Female	Malay	Degree
759	Doctors	Female	Malay	Degree
760	Doctors	Male	Malay	Degree
761	Doctors	Female	Chinese	Degree
762	Doctors	Male	Malay	Degree
763	Paramedic	Female	Malay	Diploma
764	Paramedic	Female	Malay	Diploma
765	Doctors	Male	Malay	Degree
766	Doctors	Female	Malay	Degree
767	Doctors	Male	Malay	Master
768	Paramedic	Male	Malay	Diploma
769	Doctors	Female	Malay	Degree
770	Doctors	Male	Chinese	Degree
771	Doctors	Male	Malay	Degree
772	Doctors	Female	Malay	Master
773	Doctors	Female	India	Degree
774	Paramedic	Male	Others	Diploma
775	Paramedic	Male	India	Diploma
776	Doctors	Female	Chinese	PhD / Subspecialty
777	Doctors	Male	Malay	PhD / Subspecialty
778	Doctors	Male	India	Degree
779	Doctors	Female	India	Master
780	Doctors	Female	Malay	Degree
781	Paramedic	Male	Malay	Diploma
782	Doctors	Female	Chinese	Degree
783	Doctors	Female	Malay	Degree
784	Doctors	Female	Malay	Degree
785	Doctors	Male	Malay	Degree
786	Doctors	Female	Malay	Degree

787	Doctors	Female	Malay	Degree
788	Doctors	Female	Malay	Degree
789	Paramedic	Female	Malay	Diploma
790	Doctors	Female	Malay	Degree
791	Doctors	Male	Others	Degree
792	Doctors	Female	Malay	Degree
793	Paramedic	Male	Malay	Diploma
794	Paramedic	Female	Malay	Diploma
795	Doctors	Female	Malay	Master
796	Doctors	Female	India	Degree
797	Doctors	Female	Malay	PhD / Subspecialty
798	Doctors	Male	India	Degree
799	Doctors	Male	Malay	Degree
800	Doctors	Female	Malay	Master
801	Doctors	Female	Malay	Master
802	Doctors	Female	Malay	Degree
803	Paramedic	Female	Malay	Diploma
804	Paramedic	Female	Malay	Diploma
805	Paramedic	Male	Malay	Diploma
806	Paramedic	Male	Malay	Degree
807	Paramedic	Female	Malay	Diploma
808	Technical staff	Female	Malay	Degree
809	Paramedic	Female	Malay	Diploma
810	Paramedic	Female	Malay	Secondary
811	Paramedic	Male	Malay	Diploma
812	Paramedic	Female	Malay	Diploma
813	Doctors	Female	Malay	Degree
814	Administratio	Female	Malay	Degree
815	Paramedic	Female	Malay	Degree
816	Paramedic	Female	Malay	Degree
817	Paramedic	Female	Malay	Degree
818	Doctors	Female	Malay	PhD / Subspecialty
819	Doctors	Female	Malay	Master
820	Paramedic	Female	Malay	Degree
821	Paramedic	Male	Malay	Diploma
822	Paramedic	Female	Malay	Degree
823	Doctors	Female	Malay	Master
824	Doctors	Female	Malay	Degree
825	Paramedic	Female	Malay	Diploma
826	Doctors	Male	Chinese	Master
827	Doctors	Female	Malay	Master
828	Paramedic	Female	Malay	Diploma
829	Doctors	Male	Chinese	Master
830	Administratio	Female	Malay	Diploma

831	Administratio	Female	Malay	Diploma
832	Doctors	Female	Malay	Degree
833	Doctors	Female	Malay	Master
834	Doctors	Male	Malay	Master
835	Doctors	Female	Malay	Degree
836	Doctors	Female	Malay	Degree
837	Paramedic	Female	Malay	Degree
838	Doctors	Male	Chinese	Master
839	Doctors	Female	India	Degree
840	Doctors	Male	Malay	Master
841	Doctors	Female	Malay	Master
842	Paramedic	Male	Malay	Diploma
843	Doctors	Female	India	Degree
844	Paramedic	Female	Malay	Diploma
845	Doctors	Male	Malay	Master
846	Paramedic	Female	Chinese	Master
847	Paramedic	Female	Malay	Diploma
848	Doctors	Female	Malay	Master
849	Doctors	Male	Chinese	Master
850	Doctors	Female	Malay	Degree
851	Doctors	Male	Malay	Degree
852	Doctors	Female	India	Master
853	Doctors	Female	Malay	Degree
854	Doctors	Male	Malay	PhD / Subspecialty
855	Paramedic	Male	Malay	Diploma
856	Doctors	Male	India	PhD / Subspecialty
857	Doctors	Male	Malay	Degree
858	Doctors	Female	India	Degree
859	Paramedic	Male	Malay	Diploma
860	Doctors	Female	Chinese	PhD / Subspecialty
861	Technical staff	Female	Malay	Master
862	Doctors	Male	India	Degree
863	Paramedic	Female	India	Degree
864	Paramedic	Male	Malay	Degree
865	Paramedic	Male	Malay	Degree
866	Technical staff	Male	Malay	Master
867	Technical staff	Male	Malay	Secondary
868	Doctors	Female	Malay	Degree
869	Technical staff	Male	Malay	Degree
870	Doctors	Female	Malay	Degree
871	Doctors	Female	Malay	Degree
872	Doctors	Female	Malay	Degree
873	Doctors	Male	Malay	Degree
874	Doctors	Female	Malay	Degree

875	Doctors	Male	Malay	Master
876	Doctors	Female	Malay	Master
877	Doctors	Female	Malay	Degree
878	Doctors	Female	Malay	Master
879	Doctors	Male	Malay	Master
880	Technical staff	Female	Malay	Diploma
881	Technical staff	Male	Malay	Diploma
882	Paramedic	Female	Malay	Diploma
883	Paramedic	Female	Malay	Secondary
884	Paramedic	Female	Malay	Secondary
885	Paramedic	Female	Malay	Diploma
886	Paramedic	Female	Malay	Secondary
887	Paramedic	Female	Malay	Diploma
888	Doctors	Female	Malay	Master
889	Doctors	Female	Malay	Master
890	Doctors	Male	Chinese	Degree
891	Technical staff	Female	Malay	Degree
892	Paramedic	Female	Malay	Diploma
893	Paramedic	Female	Malay	Master
894	Technical staff	Female	Malay	Degree
895	Paramedic	Female	Chinese	Diploma
896	Paramedic	Female	Chinese	Degree
897	Technical staff	Female	Malay	Degree
898	Technical staff	Male	Malay	Degree
899	Technical staff	Female	Chinese	Degree
900	Doctors	Female	Malay	Master
901	Doctors	Male	Chinese	Degree
902	Doctors	Female	Chinese	Degree
903	Doctors	Female	Chinese	Degree
904	Paramedic	Female	Malay	Diploma
905	Doctors	Female	Chinese	Degree
906	Doctors	Female	India	Degree
907	Paramedic	Male	Malay	Diploma
908	Technical staff	Female	Malay	Master
909	Doctors	Male	Malay	Master
910	Doctors	Female	Malay	Degree
911	Technical staff	Female	Malay	Degree
912	Technical staff	Female	Malay	Master
913	Administratio	Female	Chinese	Degree
914	Doctors	Male	Chinese	Degree
915	Doctors	Female	India	Degree
916	Doctors	Female	India	Degree
917	Doctors	Female	Malay	Degree
918	Technical staff	Female	Malay	Degree

919	Paramedic	Female	Malay	Diploma
920	Technical staff	Female	India	Degree
921	Technical staff	Female	Malay	Degree
922	Paramedic	Male	Malay	Degree
923	Paramedic	Female	Chinese	Degree
924	Technical staff	Male	Malay	Degree
925	Paramedic	Female	Malay	Diploma
926	Paramedic	Female	Malay	Diploma
927	Technical staff	Female	Malay	Degree
928	Paramedic	Female	Malay	Diploma
929	Administratio	Female	Malay	Secondary
930	Doctors	Female	India	Degree
931	Technical staff	Female	Malay	Degree
932	Technical staff	Female	Malay	Degree
933	Doctors	Male	India	Degree
934	Doctors	Female	Malay	Degree
935	Doctors	Female	Malay	Degree
936	Doctors	Female	Malay	Degree
937	Administratio	Female	Bumiputera	Secondary
938	Doctors	Female	Malay	Master
939	Doctors	Female	Malay	Degree
940	Paramedic	Female	Bumiputera	Degree
941	Doctors	Male	Malay	Master
942	Doctors	Male	Malay	PhD / Subspecialty
943	Technical staff	Female	Bumiputera	Diploma
944	Doctors	Male	Others	Degree
945	Doctors	Male	Bumiputera	PhD / Subspecialty
946	Administratio	Female	Malay	Degree
947	Administratio	Female	Malay	Secondary
948	Administratio	Female	Malay	Degree
949	Administratio	Female	Malay	Secondary
950	Administratio	Female	Malay	Secondary
951	Administratio	Female	Malay	Secondary
952	Administratio	Female	Malay	Secondary
953	Administratio	Female	Malay	Degree
954	Paramedic	Female	Malay	Diploma
955	Paramedic	Female	Malay	Diploma
956	Paramedic	Female	Malay	Diploma
957	Doctors	Female	Malay	Master
958	Doctors	Female	Malay	Master
959	Paramedic	Male	Malay	Diploma
960	Paramedic	Female	Malay	Diploma
961	Doctors	Female	Malay	Degree

962	Doctors	Female	Malay	Diploma
963	Doctors	Female	Bumiputera	Degree
964	Paramedic	Female	Bumiputera	Diploma
965	Paramedic	Female	Chinese	Diploma
966	Paramedic	Female	Bumiputera	Diploma
967	Paramedic	Female	Bumiputera	Diploma
968	Administratio	Female	Malay	Diploma
969	Paramedic	Female	Bumiputera	Degree
970	Paramedic	Male	Malay	Diploma
971	Paramedic	Female	Malay	Diploma
972	Doctors	Male	Chinese	Degree
973	Doctors	Male	Bumiputera	Master
974	Doctors	Male	Others	Degree
975	Technical staff	Female	Bumiputera	Diploma
976	Administratio	Male	Bumiputera	Degree
977	Technical staff	Female	Bumiputera	Diploma
978	Technical staff	Female	Bumiputera	Diploma
979	Doctors	Female	Bumiputera	Degree
980	Administratio	Male	Malay	Degree
981	Paramedic	Male	Bumiputera	Master
982	Technical staff	Female	Bumiputera	Master
983	Paramedic	Female	Bumiputera	Diploma
984	Paramedic	Female	Bumiputera	Diploma
985	Paramedic	Female	Bumiputera	Diploma
986	Paramedic	Female	Others	Diploma
987	Paramedic	Female	Bumiputera	Diploma
988	Paramedic	Male	Bumiputera	Diploma
989	Paramedic	Female	Bumiputera	Diploma
990	Paramedic	Female	Bumiputera	Diploma
991	Paramedic	Female	Bumiputera	Diploma
992	Paramedic	Female	Bumiputera	Diploma
993	Paramedic	Female	Bumiputera	Diploma
994	Paramedic	Female	Bumiputera	Degree
995	Paramedic	Female	Bumiputera	Diploma
996	Paramedic	Female	Bumiputera	Diploma
997	Technical staff	Female	Bumiputera	Diploma
998	Paramedic	Female	Bumiputera	Diploma
999	Paramedic	Female	Others	Diploma
1000	Paramedic	Female	Bumiputera	Diploma
1001	Paramedic	Female	Bumiputera	Diploma
1002	Paramedic	Male	Chinese	Master
1003	Paramedic	Female	Bumiputera	Diploma
1004	Paramedic	Female	Bumiputera	Diploma
1005	Paramedic	Female	Bumiputera	Diploma

1006	Paramedic	Female	Bumiputera	Diploma
1007	Paramedic	Female	Others	Diploma
1008	Paramedic	Female	Bumiputera	Secondary
1009	Paramedic	Female	Bumiputera	Diploma
1010	Paramedic	Male	Bumiputera	Diploma
1011	Paramedic	Female	Bumiputera	Diploma
1012	Paramedic	Female	Others	Diploma
1013	Paramedic	Female	Bumiputera	Diploma
1014	Paramedic	Female	Bumiputera	Diploma
1015	Paramedic	Male	Malay	Diploma
1016	Paramedic	Female	Bumiputera	Secondary
1017	Paramedic	Female	Bumiputera	Diploma
1018	Paramedic	Female	Bumiputera	Diploma
1019	Paramedic	Female	Bumiputera	Diploma
1020	Administratio	Male	Bumiputera	Degree
1021	Paramedic	Female	Bumiputera	Diploma
1022	Paramedic	Female	Others	Diploma
1023	Paramedic	Female	Bumiputera	Diploma
1024	Paramedic	Female	Bumiputera	Diploma
1025	Paramedic	Female	Bumiputera	Diploma
1026	Paramedic	Female	Bumiputera	Diploma
1027	Paramedic	Female	Bumiputera	Diploma
1028	Paramedic	Female	Bumiputera	Diploma
1029	Paramedic	Female	Others	Diploma
1030	Paramedic	Female	Bumiputera	Diploma
1031	Paramedic	Female	Malay	Diploma
1032	Administratio	Male	Bumiputera	Master
1033	Paramedic	Female	Bumiputera	Diploma
1034	Administratio	Female	Bumiputera	Diploma
1035	Paramedic	Female	Bumiputera	Diploma
1036	Doctors	Female	Others	Degree
1037	Doctors	Female	Malay	Secondary
1038	Paramedic	Female	Bumiputera	Diploma
1039	Paramedic	Female	Bumiputera	Diploma
1040	Paramedic	Female	Malay	Diploma
1041	Administratio	Female	Malay	Diploma
1042	Doctors	Female	Malay	Degree
1043	Doctors	Female	Malay	Degree
1044	Paramedic	Male	Bumiputera	Diploma
1045	Paramedic	Female	Others	Diploma
1046	Paramedic	Female	Bumiputera	Diploma
1047	Technical staff	Female	Bumiputera	Diploma
1048	Paramedic	Female	Malay	Diploma
1049	Doctors	Female	Chinese	Degree

1050	Paramedic	Female	Malay	Diploma
1051	Doctors	Male	Malay	Secondary
1052	Doctors	Female	Malay	Degree
1053	Doctors	Female	Chinese	PhD / Subspecialty
1054	Doctors	Male	India	Master
1055	Doctors	Female	Malay	Master
1056	Doctors	Male	Malay	Degree
1057	Paramedic	Male	Malay	Diploma
1058	Technical staff	Male	Malay	Secondary
1059	Doctors	Male	Chinese	Degree
1060	Paramedic	Male	Malay	Diploma
1061	Doctors	Female	Malay	Degree
1062	Paramedic	Male	Malay	Secondary
1063	Doctors	Male	Malay	Degree
1064	Paramedic	Female	Malay	Diploma
1065	Paramedic	Female	Malay	Diploma
1066	Doctors	Female	Malay	Degree
1067	Doctors	Male	Chinese	Master
1068	Doctors	Female	Malay	Diploma
1069	Administratio	Female	Malay	Diploma
1070	Paramedic	Female	Malay	Diploma
1071	Paramedic	Female	Malay	Diploma
1072	Paramedic	Female	Malay	Diploma
1073	Paramedic	Female	Malay	Degree
1074	Technical staff	Female	Chinese	Degree
1075	Technical staff	Female	India	Diploma
1076	Technical staff	Female	Malay	Master
1077	Doctors	Male	Malay	Degree
1078	Paramedic	Female	Malay	Diploma
1079	Paramedic	Female	Malay	Degree
1080	Doctors	Female	Chinese	Degree
1081	Paramedic	Female	Malay	Diploma
1082	Paramedic	Male	Malay	Master
1083	Doctors	Female	India	Degree
1084	Doctors	Female	Chinese	Secondary
1085	Administratio	Female	Malay	Master
1086	Doctors	Female	Malay	PhD / Subspecialty
1087	Technical staff	Female	Malay	Degree
1088	Doctors	Male	Chinese	PhD / Subspecialty
1089	Technical staff	Female	Malay	Diploma
1090	Paramedic	Female	Chinese	Diploma
1091	Technical staff	Male	Malay	Degree
1092	Paramedic	Female	Malay	Diploma
1093	Paramedic	Male	Malay	Degree

1094	Doctors	Female	Malay	Degree
1095	Doctors	Male	Malay	Degree
1096	Doctors	Male	Malay	Master
1097	Paramedic	Male	Malay	Secondary
1098	Paramedic	Female	Malay	Diploma
1099	Paramedic	Female	Malay	Diploma
1100	Paramedic	Female	Malay	Diploma
1101	Paramedic	Female	Malay	Diploma
1102	Paramedic	Male	Malay	Diploma
1103	Doctors	Female	Malay	Degree
1104	Paramedic	Male	Malay	Diploma
1105	Paramedic	Male	Malay	Diploma
1106	Paramedic	Female	Bumiputera	Diploma
1107	Doctors	Male	Malay	Master
1108	Administratio	Female	Malay	Diploma
1109	Paramedic	Male	Malay	Master
1110	Doctors	Female	Malay	Degree
1111	Doctors	Female	Malay	Degree
1112	Technical staff	Female	Chinese	Degree
1113	Paramedic	Male	Malay	Diploma
1114	Paramedic	Female	Malay	Degree
1115	Doctors	Female	Malay	Master
1116	Doctors	Female	Malay	PhD / Subspecialty
1117	Paramedic	Female	Malay	Diploma
1118	Paramedic	Female	Malay	Diploma
1119	Paramedic	Female	Malay	Diploma
1120	Paramedic	Female	Malay	Diploma
1121	Doctors	Male	Bumiputera	Degree
1122	Paramedic	Female	Malay	Diploma
1123	Doctors	Female	Malay	Degree
1124	Doctors	Female	Malay	Degree
1125	Pharmacist	Female	Malay	Master
1126	Pharmacist	Female	Malay	Master
1127	Pharmacist	Female	Malay	Degree
1128	Pharmacist	Female	Chinese	Master
1129	Pharmacist	Male	Malay	PhD / Subspecialty
1130	Pharmacist	Male	Chinese	Degree
1131	Pharmacist	Female	India	Degree
1132	Pharmacist	Female	Chinese	Degree
1133	Doctors	Male	Malay	Degree
1134	Doctors	Male	Malay	Degree
1135	Doctors	Female	Malay	Degree
1136	Paramedic	Male	Malay	Diploma
1137	Doctors	Male	Malay	Degree

1138	Doctors	Male	Chinese	Degree
1139	Administratio	Female	Malay	Diploma
1140	Doctors	Male	Malay	Degree
1141	Doctors	Male	Chinese	Master
1142	Doctors	Female	Malay	Degree
1143	Paramedic	Female	Malay	Degree
1144	Paramedic	Male	Malay	Diploma
1145	Pharmacist	Female	Malay	Degree
1146	Paramedic	Male	Malay	Diploma
1147	Doctors	Female	Malay	Degree
1148	Doctors	Female	Malay	Degree
1149	Paramedic	Female	Malay	Diploma
1150	Administratio	Female	Malay	Diploma
1151	Pharmacist	Female	Chinese	Degree
1152	Pharmacist	Male	Chinese	Degree
1153	Pharmacist	Female	Malay	Degree
1154	Paramedic	Male	Malay	Degree
1155	Pharmacist	Female	Malay	Degree
1156	Pharmacist	Female	Malay	Degree
1157	Pharmacist	Female	Malay	Degree
1158	Pharmacist	Female	Malay	Degree
1159	Doctors	Female	Malay	Master
1160	Paramedic	Female	Bumiputera	Diploma
1161	Doctors	Male	Malay	Degree
1162	Doctors	Female	Malay	Degree
1163	Doctors	Female	Malay	Degree
1164	Pharmacist	Female	Malay	Degree
1165	Doctors	Male	Malay	Degree
1166	Doctors	Female	Malay	Master
1167	Paramedic	Male	Malay	Master
1168	Doctors	Male	Malay	Degree
1169	Doctors	Male	Chinese	Degree
1170	Doctors	Female	Malay	Degree
1171	Doctors	Female	Malay	Degree
1172	Doctors	Female	Malay	Master
1173	Doctors	Female	Malay	Degree
1174	Doctors	Male	Malay	Master
1175	Doctors	Female	Malay	Degree
1176	Doctors	Female	Malay	Degree
1177	Doctors	Female	Malay	Master
1178	Doctors	Female	Malay	Degree
1179	Doctors	Male	Malay	Degree
1180	Doctors	Male	Malay	Degree
1181	Doctors	Female	Malay	PhD / Subspecialty

1182	Doctors	Female	Malay	Degree
1183	Doctors	Female	Chinese	Degree
1184	Doctors	Female	Malay	Degree
1185	Doctors	Male	Malay	Degree
1186	Doctors	Female	Malay	Degree
1187	Doctors	Female	Malay	Master
1188	Paramedic	Female	Chinese	Diploma
1189	Doctors	Male	Malay	Master
1190	Doctors	Female	India	Degree
1191	Doctors	Female	Malay	Degree
1192	Doctors	Female	Malay	Degree
1193	Doctors	Male	Malay	Master
1194	Doctors	Male	Malay	Degree
1195	Doctors	Female	India	Degree
1196	Technical staff	Female	India	Diploma
1197	Doctors	Male	India	Degree
1198	Doctors	Male	India	Degree
1199	Doctors	Female	India	PhD / Subspecialty
1200	Doctors	Female	Others	Degree
1201	Administratio	Male	Malay	Diploma
1202	Doctors	Male	India	Degree
1203	Paramedic	Male	Malay	Diploma
1204	Paramedic	Female	Malay	Diploma
1205	Doctors	Male	Chinese	Degree
1206	Doctors	Female	Malay	Degree
1207	Doctors	Female	India	Degree
1208	Doctors	Male	Malay	Degree
1209	Doctors	Female	Chinese	Degree
1210	Doctors	Male	Chinese	Degree
1211	Doctors	Female	India	Degree
1212	Paramedic	Female	Malay	Degree
1213	Doctors	Male	Malay	Degree
1214	Doctors	Male	Malay	Degree
1215	Doctors	Female	India	Degree
1216	Doctors	Female	Malay	Degree
1217	Doctors	Female	India	PhD / Subspecialty
1218	Doctors	Male	Malay	Degree
1219	Doctors	Male	Malay	Master
1220	Doctors	Female	India	Degree
1221	Doctors	Female	Malay	Degree
1222	Doctors	Male	Malay	Master
1223	Doctors	Male	Malay	Degree
1224	Doctors	Male	Malay	Master
1225	Doctors	Female	Chinese	Degree

1226	Paramedic	Female	Malay	Diploma
1227	Doctors	Male	Malay	Master
1228	Paramedic	Female	Bumiputera	Diploma
1229	Doctors	Female	Malay	Master
1230	Doctors	Female	Malay	Master
1231	Doctors	Male	Chinese	Degree
1232	Doctors	Female	Chinese	Master
1233	Doctors	Male	Chinese	Master
1234	Doctors	Female	Malay	PhD / Subspecialty
1235	Doctors	Male	Chinese	Degree
1236	Doctors	Male	India	Degree
1237	Doctors	Female	Bumiputera	Degree
1238	Paramedic	Female	Malay	Diploma
1239	Paramedic	Female	Malay	Diploma
1240	Paramedic	Female	Malay	Diploma
1241	Paramedic	Female	Bumiputera	Diploma
1242	Paramedic	Female	Malay	Diploma
1243	Doctors	Female	Malay	Secondary
1244	Doctors	Male	Malay	Degree
1245	Doctors	Female	India	Degree
1246	Doctors	Female	Malay	Degree
1247	Paramedic	Female	India	Degree
1248	Paramedic	Female	Malay	Diploma
1249	Paramedic	Female	Malay	Diploma
1250	Doctors	Male	Malay	Degree
1251	Administratio	Female	Malay	Degree
1252	Administratio	Male	Malay	Master
1253	Administratio	Female	Malay	Diploma
1254	Paramedic	Male	Malay	Diploma
1255	Paramedic	Male	Malay	Diploma
1256	Administratio	Male	Malay	Diploma
1257	Administratio	Female	Malay	Degree
1258	Technical staff	Male	Malay	Degree
1259	Administratio	Female	Malay	Secondary
1260	Doctors	Male	Malay	Master
1261	Paramedic	Male	Malay	Degree
1262	Administratio	Female	Chinese	Secondary
1263	Paramedic	Female	Malay	Degree
1264	Paramedic	Female	Malay	Diploma
1265	Paramedic	Male	Malay	Diploma
1266	Paramedic	Male	Malay	Diploma
1267	Administratio	Female	Malay	Degree
1268	Administratio	Female	Malay	Degree
1269	Paramedic	Male	Malay	Diploma

1270	Paramedic	Male	Malay	Diploma
1271	Paramedic	Male	Malay	Degree
1272	Paramedic	Male	Bumiputera	Diploma
1273	Paramedic	Male	Malay	Diploma
1274	Paramedic	Female	Malay	Diploma
1275	Doctors	Female	India	Master
1276	Technical staff	Female	Malay	Degree
1277	Doctors	Female	India	Master
1278	Administratio	Male	Malay	Diploma
1279	Paramedic	Male	Malay	Diploma
1280	Doctors	Female	Malay	Degree
1281	Paramedic	Female	Malay	Diploma
1282	Paramedic	Female	Bumiputera	Diploma
1283	Paramedic	Female	Malay	Diploma
1284	Paramedic	Female	Bumiputera	Diploma
1285	Paramedic	Female	Malay	Diploma
1286	Paramedic	Female	India	Diploma
1287	Paramedic	Female	Malay	Diploma
1288	Paramedic	Female	Malay	Diploma
1289	Paramedic	Male	Chinese	Diploma
1290	Paramedic	Female	Bumiputera	Diploma
1291	Paramedic	Female	Malay	Diploma
1292	Paramedic	Male	Malay	Diploma
1293	Paramedic	Female	India	Degree
1294	Paramedic	Female	India	Degree
1295	Paramedic	Female	Malay	Diploma
1296	Paramedic	Female	Bumiputera	Diploma
1297	Paramedic	Female	Malay	Diploma
1298	Paramedic	Male	Malay	Diploma
1299	Paramedic	Female	Malay	Diploma
1300	Paramedic	Male	Malay	Diploma
1301	Paramedic	Female	Malay	Diploma
1302	Paramedic	Female	Malay	Diploma
1303	Paramedic	Female	India	Diploma
1304	Paramedic	Female	Bumiputera	Diploma
1305	Paramedic	Female	Malay	Diploma
1306	Paramedic	Female	Malay	Degree
1307	Paramedic	Female	Malay	Diploma
1308	Paramedic	Female	Malay	Degree
1309	Paramedic	Female	Malay	Diploma
1310	Paramedic	Female	Malay	Diploma
1311	Paramedic	Female	Malay	Diploma
1312	Paramedic	Female	Malay	Diploma
1313	Paramedic	Female	Malay	Diploma

1314	Technical staff	Female	Malay	Diploma
1315	Pharmacist	Female	India	Degree
1316	Paramedic	Female	India	Diploma
1317	Administratio	Female	Malay	Master
1318	Paramedic	Female	Others	Diploma
1319	Paramedic	Female	India	Diploma
1320	Doctors	Female	Malay	Degree
1321	Pharmacist	Female	Chinese	Degree
1322	Paramedic	Female	Malay	Diploma
1323	Doctors	Male	Chinese	Degree
1324	Pharmacist	Female	Malay	Degree
1325	Administratio	Female	Malay	Diploma
1326	Paramedic	Female	Malay	Diploma
1327	Doctors	Female	Malay	Degree
1328	Doctors	Male	Malay	Master
1329	Doctors	Male	Chinese	Master
1330	Paramedic	Male	Malay	Diploma
1331	Paramedic	Male	Malay	Diploma
1332	Doctors	Female	Chinese	Degree
1333	Doctors	Female	Malay	Degree
1334	Technical staff	Female	Malay	Diploma
1335	Paramedic	Male	Malay	Diploma
1336	Doctors	Male	Malay	Master
1337	Doctors	Male	Malay	Degree
1338	Paramedic	Male	Malay	Diploma
1339	Technical staff	Female	Malay	Diploma
1340	Doctors	Female	India	Master
1341	Paramedic	Female	Malay	Diploma
1342	Doctors	Female	Malay	Degree
1343	Doctors	Male	Malay	Degree
1344	Administratio	Female	Malay	Diploma
1345	Technical staff	Female	Malay	Diploma
1346	Administratio	Female	Malay	Diploma
1347	Technical staff	Female	Malay	Diploma
1348	Technical staff	Male	Malay	Diploma
1349	Paramedic	Female	Malay	Degree
1350	Doctors	Male	Malay	Degree
1351	Doctors	Female	India	Master
1352	Administratio	Female	Malay	Secondary
1353	Doctors	Female	Malay	Degree
1354	Administratio	Female	Malay	Diploma
1355	Doctors	Female	Malay	Degree
1356	Administratio	Female	Malay	Diploma
1357	Paramedic	Female	Malay	Degree

1358	Paramedic	Female	Malay	Diploma
1359	Paramedic	Female	Malay	Diploma
1360	Paramedic	Female	Malay	Diploma
1361	Paramedic	Female	Malay	Diploma
1362	Paramedic	Female	Malay	Diploma
1363	Paramedic	Female	Malay	Diploma
1364	Paramedic	Female	Malay	Diploma
1365	Paramedic	Female	Malay	Diploma
1366	Paramedic	Female	Malay	Diploma
1367	Paramedic	Female	Malay	Diploma
1368	Paramedic	Female	Malay	Secondary
1369	Paramedic	Female	Chinese	Diploma
1370	Administratio	Female	Malay	Diploma
1371	Doctors	Male	Malay	Degree
1372	Paramedic	Female	Malay	Diploma
1373	Paramedic	Female	Malay	Secondary
1374	Paramedic	Female	Malay	Diploma
1375	Pharmacist	Male	Chinese	Degree
1376	Administratio	Female	Malay	Diploma
1377	Paramedic	Male	Malay	Diploma
1378	Doctors	Female	Malay	Degree
1379	Doctors	Male	Malay	Degree
1380	Doctors	Female	Chinese	Degree
1381	Doctors	Male	Malay	Degree
1382	Doctors	Male	Malay	Degree
1383	Doctors	Female	Others	Degree
1384	Doctors	Female	Malay	Degree
1385	Doctors	Female	Malay	Master
1386	Doctors	Female	Chinese	Master
1387	Doctors	Female	India	Master
1388	Paramedic	Male	Malay	Diploma
1389	Doctors	Male	Malay	Degree
1390	Administratio	Female	Malay	Secondary
1391	Paramedic	Female	Malay	Diploma
1392	Paramedic	Male	Malay	Diploma
1393	Doctors	Female	Malay	Degree
1394	Doctors	Female	Malay	Degree
1395	Doctors	Male	Malay	Degree
1396	Paramedic	Female	Malay	Diploma
1397	Doctors	Female	Malay	Degree
1398	Technical staff	Female	India	Secondary
1399	Paramedic	Female	Malay	Diploma
1400	Paramedic	Female	Malay	Diploma
1401	Paramedic	Female	Malay	Diploma

1402	Paramedic	Female	Malay	Secondary
1403	Paramedic	Female	Malay	Secondary
1404	Doctors	Female	Malay	Degree
1405	Paramedic	Male	Malay	Diploma
1406	Paramedic	Female	Malay	Diploma
1407	Paramedic	Female	Malay	Diploma
1408	Paramedic	Female	Malay	Diploma
1409	Paramedic	Female	Malay	Diploma
1410	Doctors	Female	Malay	Degree
1411	Doctors	Female	Malay	Degree
1412	Doctors	Male	Malay	Degree
1413	Doctors	Male	Malay	Degree
1414	Doctors	Male	Malay	Degree
1415	Paramedic	Male	Malay	Diploma
1416	Paramedic	Female	Malay	Diploma
1417	Paramedic	Female	Malay	Degree
1418	Doctors	Female	Malay	Degree
1419	Doctors	Female	Malay	Degree
1420	Paramedic	Female	Malay	Diploma
1421	Paramedic	Female	Malay	Diploma
1422	Doctors	Female	Malay	Master
1423	Doctors	Female	Chinese	Master
1424	Paramedic	Male	Malay	Diploma
1425	Paramedic	Female	Malay	Diploma
1426	Doctors	Male	Malay	Degree
1427	Administratio	Female	Malay	Degree
1428	Administratio	Female	Malay	Degree
1429	Doctors	Female	Malay	Master
1430	Doctors	Female	Malay	Degree
1431	Doctors	Male	Malay	Degree
1432	Technical staff	Male	Malay	Diploma
1433	Pharmacist	Female	Malay	Degree
1434	Pharmacist	Female	Malay	Degree
1435	Doctors	Male	Malay	Degree
1436	Paramedic	Female	Chinese	Diploma
1437	Paramedic	Female	Bumiputera	Diploma
1438	Paramedic	Female	Bumiputera	Diploma
1439	Administratio	Female	Malay	Diploma
1440	Paramedic	Female	Malay	Diploma
1441	Administratio	Female	Malay	Degree
1442	Doctors	Male	Chinese	Master
1443	Doctors	Female	India	Degree
1444	Doctors	Female	Malay	Degree
1445	Doctors	Female	India	Degree

1446	Doctors	Female	India	Degree
1447	Doctors	Female	India	PhD / Subspecialty
1448	Doctors	Female	Malay	Degree
1449	Paramedic	Male	Malay	Diploma
1450	Paramedic	Female	Chinese	Diploma
1451	Doctors	Male	Chinese	Master
1452	Pharmacist	Female	Chinese	Degree
1453	Pharmacist	Female	Malay	Degree
1454	Doctors	Male	India	Degree
1455	Administratio	Female	Malay	Diploma
1456	Pharmacist	Female	India	Degree
1457	Pharmacist	Female	Malay	Degree
1458	Doctors	Female	India	PhD / Subspecialty
1459	Technical staff	Male	Malay	Diploma
1460	Administratio	Female	Malay	Diploma
1461	Administratio	Male	India	Diploma
1462	Doctors	Female	Chinese	Master
1463	Doctors	Male	India	Degree
1464	Doctors	Female	Chinese	Degree
1465	Doctors	Female	Bumiputera	PhD / Subspecialty
1466	Doctors	Male	Chinese	Master
1467	Doctors	Female	India	Master
1468	Paramedic	Male	Malay	Diploma
1469	Paramedic	Male	Malay	Diploma
1470	Doctors	Female	Chinese	Master
1471	Doctors	Female	India	Degree
1472	Doctors	Female	India	Degree
1473	Technical staff	Male	Malay	Degree
1474	Technical staff	Male	India	Degree
1475	Paramedic	Male	Malay	Diploma
1476	Doctors	Male	Malay	Degree
1477	Doctors	Male	India	Master
1478	Doctors	Female	Malay	Degree
1479	Doctors	Male	India	PhD / Subspecialty
1480	Doctors	Female	Chinese	Master
1481	Doctors	Female	Malay	Master
1482	Doctors	Male	Malay	Degree
1483	Paramedic	Male	Malay	Secondary
1484	Doctors	Male	Malay	Master
1485	Administratio	Female	Malay	Secondary
1486	Doctors	Female	Malay	Degree
1487	Doctors	Male	Malay	Master
1488	Doctors	Male	Malay	Degree
1489	Doctors	Female	Malay	Degree

1490	Doctors	Male	Malay	Master
1491	Doctors	Male	Malay	Master
1492	Doctors	Female	Chinese	Degree
1493	Paramedic	Male	Malay	Diploma
1494	Doctors	Male	Malay	Degree
1495	Paramedic	Male	Bumiputera	Diploma
1496	Doctors	Female	India	Master
1497	Administratio	Female	Malay	Diploma
1498	Doctors	Male	Malay	Degree
1499	Paramedic	Female	India	Degree
1500	Paramedic	Female	Malay	Diploma
1501	Paramedic	Female	Malay	Diploma
1502	Administratio	Female	Malay	Diploma
1503	Paramedic	Female	Malay	Diploma
1504	Paramedic	Female	Malay	Diploma
1505	Paramedic	Female	Malay	Diploma
1506	Doctors	Male	Malay	Master
1507	Paramedic	Female	Malay	Diploma
1508	Paramedic	Female	Malay	Diploma
1509	Paramedic	Female	Malay	Diploma
1510	Paramedic	Female	Malay	Diploma
1511	Doctors	Male	Chinese	Master
1512	Doctors	Male	Malay	Degree
1513	Doctors	Female	Malay	Degree
1514	Doctors	Female	Malay	Degree
1515	Doctors	Male	Chinese	Degree
1516	Technical staff	Female	Malay	Diploma
1517	Doctors	Female	Malay	Master
1518	Doctors	Female	India	Degree
1519	Doctors	Male	Chinese	Degree
1520	Doctors	Female	Malay	Degree
1521	Doctors	Female	Malay	Degree
1522	Paramedic	Female	Bumiputera	Diploma
1523	Paramedic	Female	Malay	Diploma
1524	Paramedic	Female	Malay	Diploma
1525	Paramedic	Female	Malay	Diploma
1526	Doctors	Female	Chinese	Master
1527	Doctors	Male	Malay	Degree
1528	Doctors	Male	Chinese	Degree
1529	Doctors	Male	Others	Degree
1530	Paramedic	Female	Malay	Diploma
1531	Paramedic	Female	Malay	Diploma
1532	Paramedic	Male	Malay	Diploma
1533	Doctors	Female	India	Degree

1534	Doctors	Male	Chinese	Degree
1535	Paramedic	Female	Malay	Diploma
1536	Paramedic	Female	Malay	Diploma
1537	Paramedic	Female	Malay	Degree
1538	Paramedic	Female	Malay	Degree
1539	Paramedic	Female	Malay	Diploma
1540	Paramedic	Female	Malay	Degree
1541	Paramedic	Female	Chinese	Degree
1542	Paramedic	Female	Malay	Secondary
1543	Paramedic	Female	Malay	Degree
1544	Paramedic	Female	India	Diploma
1545	Paramedic	Female	Malay	Diploma
1546	Paramedic	Female	Malay	Secondary
1547	Doctors	Female	Malay	Degree
1548	Paramedic	Female	Malay	Diploma
1549	Doctors	Male	Malay	Master
1550	Administratio	Female	India	Master
1551	Paramedic	Female	Malay	Diploma
1552	Paramedic	Female	Malay	Diploma
1553	Paramedic	Female	Malay	Diploma
1554	Paramedic	Female	Malay	Diploma
1555	Paramedic	Female	Malay	Diploma
1556	Technical staff	Female	Malay	Diploma
1557	Paramedic	Female	Malay	Diploma
1558	Paramedic	Female	Malay	Diploma
1559	Paramedic	Female	Malay	Diploma
1560	Paramedic	Female	Malay	Diploma
1561	Paramedic	Female	Malay	Diploma
1562	Pharmacist	Female	Malay	Master
1563	Paramedic	Female	Malay	Diploma
1564	Paramedic	Female	Malay	Diploma
1565	Paramedic	Female	Malay	Diploma
1566	Doctors	Male	Malay	Master
1567	Paramedic	Female	Malay	Diploma
1568	Paramedic	Female	Malay	Diploma
1569	Paramedic	Female	Malay	Diploma
1570	Paramedic	Female	Malay	Diploma
1571	Paramedic	Female	Malay	Diploma
1572	Paramedic	Female	Others	Degree
1573	Paramedic	Female	Malay	Diploma
1574	Paramedic	Female	Bumiputera	Diploma
1575	Paramedic	Female	Malay	Diploma
1576	Paramedic	Female	Bumiputera	Diploma
1577	Paramedic	Female	Malay	Diploma

1578	Paramedic	Female	Bumiputera	Diploma
1579	Paramedic	Female	Malay	Diploma
1580	Paramedic	Female	Malay	Diploma
1581	Paramedic	Female	Malay	Diploma
1582	Paramedic	Female	Malay	Diploma
1583	Paramedic	Female	Malay	Diploma
1584	Paramedic	Female	Malay	Diploma
1585	Paramedic	Female	Malay	Diploma
1586	Paramedic	Female	Malay	Diploma
1587	Paramedic	Female	Malay	Diploma
1588	Paramedic	Female	Malay	Diploma
1589	Paramedic	Female	Malay	Diploma
1590	Paramedic	Female	Malay	Diploma
1591	Paramedic	Male	Malay	Diploma
1592	Paramedic	Female	Malay	Diploma
1593	Doctors	Female	Chinese	PhD / Subspecialty
1594	Paramedic	Female	Malay	Diploma
1595	Paramedic	Female	Malay	Diploma
1596	Paramedic	Female	Malay	Diploma
1597	Paramedic	Female	Malay	Diploma
1598	Paramedic	Female	Malay	Diploma
1599	Paramedic	Female	India	Diploma
1600	Paramedic	Female	Malay	Diploma
1601	Paramedic	Female	Malay	Diploma
1602	Paramedic	Female	Malay	Diploma
1603	Paramedic	Female	Malay	Diploma
1604	Doctors	Female	Malay	Degree
1605	Paramedic	Female	Malay	Diploma
1606	Paramedic	Female	Malay	Diploma
1607	Paramedic	Female	Malay	Diploma
1608	Administratio	Female	Malay	Diploma
1609	Paramedic	Female	Malay	Diploma
1610	Paramedic	Female	Malay	Diploma
1611	Paramedic	Female	Malay	Diploma
1612	Paramedic	Female	Malay	Diploma
1613	Paramedic	Female	Malay	Diploma
1614	Paramedic	Female	Malay	Diploma
1615	Paramedic	Female	Malay	Diploma
1616	Paramedic	Female	Malay	Diploma
1617	Paramedic	Male	Malay	Diploma
1618	Paramedic	Female	Malay	Diploma
1619	Paramedic	Female	Malay	Diploma
1620	Doctors	Female	Malay	Degree
